# Homologous recombination deficiency derived from whole-genome sequencing predicts platinum response in triple-negative breast cancers

**DOI:** 10.1038/s41467-023-37537-2

**Published:** 2023-04-07

**Authors:** Petra ter Brugge, Sarah C. Moser, Ivan Bièche, Petra Kristel, Sabrina Ibadioune, Alexandre Eeckhoutte, Roebi de Bruijn, Eline van der Burg, Catrin Lutz, Stefano Annunziato, Julian de Ruiter, Julien Masliah Planchon, Sophie Vacher, Laura Courtois, Rania El-Botty, Ahmed Dahmani, Elodie Montaudon, Ludivine Morisset, Laura Sourd, Léa Huguet, Heloise Derrien, Fariba Nemati, Sophie Chateau-Joubert, Thibaut Larcher, Anne Salomon, Didier Decaudin, Fabien Reyal, Florence Coussy, Tatiana Popova, Jelle Wesseling, Marc-Henri Stern, Jos Jonkers, Elisabetta Marangoni

**Affiliations:** 1 Division of Molecular Pathology, Oncode Institute, The Netherlands Cancer Institute, Amsterdam, Netherlands; 2 Genetics Department, Institut Curie, PSL University, 26 Rue d’Ulm, 75005 Paris, France; 3 INSERM U830, Institut Curie, PSL University, 75005 Paris, France; 4 Institut Curie, PSL University, 26 Rue d’Ulm, 75005 Paris, France; 5 Laboratory of Preclinical Investigation, Translational Research Department, Institut Curie, PSL University, 26 Rue d’Ulm, 75005 Paris, France; 6 BioPôle Alfort, Ecole Nationale Vétérinaire d’Alfort, 94704 Maisons Alfort, France; 7 INRA, APEX-PAnTher, Oniris, 44300 Nantes, France; 8 Department of Pathology, Institut Curie, PSL University, 75005 Paris, France; 9 Department of Surgery, Institut Curie, PSL University, 75005 Paris, France; 10 Department of Medical Oncology, Institut Curie, PSL University, 75005 Paris, France

**Keywords:** Health sciences, Breast cancer

## Abstract

The high frequency of homologous recombination deficiency (HRD) is the main rationale of testing platinum-based chemotherapy in triple-negative breast cancer (TNBC), however, the existing methods to identify HRD are controversial and there is a medical need for predictive biomarkers. We assess the in vivo response to platinum agents in 55 patient-derived xenografts (PDX) of TNBC to identify determinants of response. The HRD status, determined from whole genome sequencing, is highly predictive of platinum response. *BRCA1* promoter methylation is not associated with response, in part due to residual *BRCA1* gene expression and homologous recombination proficiency in different tumours showing mono-allelic methylation. Finally, in 2 cisplatin sensitive tumours we identify mutations in *XRCC3* and *ORC1* genes that are functionally validated in vitro. In conclusion, our results demonstrate that the genomic HRD is predictive of platinum response in a large cohort of TNBC PDX and identify alterations in *XRCC3* and *ORC1* genes driving cisplatin response.

## Abstract (web summary)

Homologous recombination deficiency is linked with platinum-based chemotherapy response in triple-negative breast cancer (TNBC) but methods to clinically identify these patients are lacking. Here, using patient-derived xenografts of TNBC the authors demonstrate that shallow HRD is predictive of response to platinum-based chemotherapy.

## Introduction

About 15% of women who develop breast cancer are diagnosed with triple-negative breast cancer (TNBC)^1^. TNBC is a heterogeneous disease, with a substantial percentage of tumours showing homologous recombination deficiency (HRD) as a result of mutations in the *BRCA1* and *BRCA2* genes^1–3^. This may sensitize tumours to DNA double-strand break (DSB) inducing therapeutic agents, such as PARP inhibitors or platinum salts. Indeed, both preclinical^4,5^ and clinical^3,6,7^ studies have shown sensitivity to DSB inducing agents in *BRCA1/2* mutated cells and tumours. A similar sensitivity may be found in tumours with molecular changes that mimic the *BRCA* mutated phenotype, such as *BRCA1* epigenetic inactivation or mutations in other genes involved in DNA DSB repair^2,5,8^. This greatly increases the percentage of patients with TNBC who may benefit from DSB inducing therapeutic agents^3^.

Platinum salts (e.g., carboplatin, cisplatin) are old drugs still used to treat TNBC in the neo-adjuvant setting, where they are added to anthracycline and taxane-based chemotherapy, and in the metastatic setting where they are generally given as first line treatment. However, only a subset of patients respond to these drugs, raising the question of how to select them properly and avoid toxicity for patients unlikely to respond.

Several strategies have been developed to distinguish BRCA-like from non-BRCA-like tumours, including detection of *BRCA1* mutation, genomic scar assays or functional testing of HR capacity by measurement of RAD51 foci^9,10^. Different clinical trials have tested the predictive value of these methods in predicting response to platinum in early and advanced settings^11^, however, results have been discordant and predictive biomarkers for platinum salts in TNBC still represent an unmet clinical need. One of the difficulties in interpreting clinical trials results lies in the fact that platinum drugs were tested in combinations with other chemotherapies inducing DNA damage, including anthracyclines and cyclophosphamide, that contribute to patients’ treatment response. A second issue that emerged from clinical trials in the advanced setting, is that HRD was evaluated on archival breast cancer tissues that do not always reflect the tumour status at the time of platinum response.

In this work, we analyse the correlation between platinum response and different predictive biomarkers, including *BRCA1/2* mutations, *BRCA1* methylation, genomic and functional signature of HRD, in a large panel of TNBC PDX. Moreover, some of these tumours are sequenced by whole-exome sequencing (WES) with their matched patients’ tumours to identify potential determinants of platinum response that are functionally validated in cell lines.

## Results

### Response to platinum chemotherapy in a large cohort of TNBC PDX models

We evaluate the response to platinum-based chemotherapy in a cohort of 55 PDX models of early TNBC, established at the Institut Curie^12,13^. Patient’s clinical characteristics are summarized in Table 1 and in Supplementary Data 1 with PDX molecular characteristics.

**Table 1:** Clinical characteristics of TNBC patients

		*n* (%)
Mean age at diagnosis (range)		54 (29–88)
TNM	T0	0 (0)
	T1	15 (29.1)
	T2	31 (54.5)
	T3	9 (16.4)
	T4	0 (0)
	N0	36 (65.4)
	N1	10 (18.2)
	N2	7 (12.7)
	N3	2 (3.6)
	M0	51 (92.7)
	M1	4 (7.3)
Breast surgery (*n* = 55)	Tumorectomy	32 (58.1)
	Mastectomy	23 (41.8)
Nodes surgery (*n* = 50)	Sentinel node biopsy	9 (18)
	Lymphadenectomy	41 (82)
Histologic type	No special type	49 (89.1)
	Metaplastic	6 (10.9)
SBR grade (*n* = 43)	Grade SBR 1	0 (0)
	Grade SBR 2	2 (4.7)
	Grade SBR 3	41 (95.3)
Type of chemotherapy (*n* = 46)	Anthracycline then taxane	30 (55.6)
	Anthracycline based	10 (18.5)
	Taxane based	4 (7.4)
	Others	2 (3.7)
	No	8 (14.8)
Radiotherapy	Yes	48 (87.3)
	No	7 (12.7)
Relapse	No	25 (45.5)
	Local only	1 (1.8)
	Distant metastasis	29 (52.7)

TNM: classification of malignant tumours (tumour, lymph node, metastasis). SBR grade: Scarff Bloom and Richardson.

This cohort of 55 patients presented the usual characteristics of TNBC^14^. Mean age at diagnosis was 54 years. TNM staging mostly corresponded to T2 (55%), N0 (65%) with a small percentage of synchronous metastasis (7%). Histologically, 89% of tumours were invasive ductal carcinoma of no special type (NST) and 11% were metaplastic carcinomas. Ninety-five percent of patients had a high SBR histological grade. The majority of patients received sequential chemotherapy (anthracycline then taxane) as adjuvant or neoadjuvant therapy. Fifty-three percent of patients developed distant metastases.

**Fig. 1:**
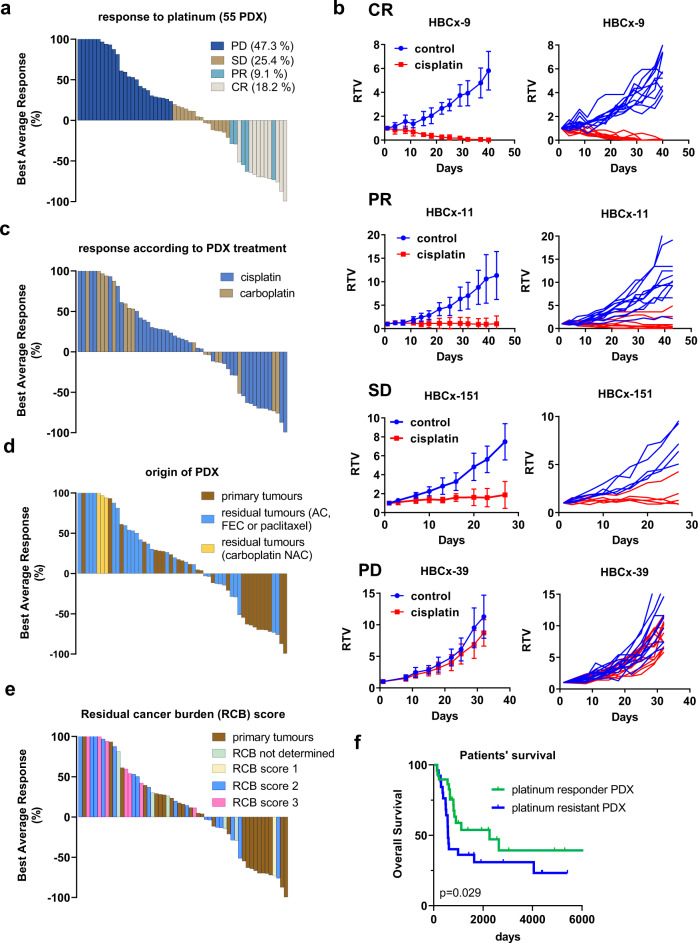
Response to platinum-based chemotherapy in the first cohort of PDX. **a** Waterfall plot representing platinum response in 55 TNBC PDX. Each bar represents the median best average response of treated xenografts from a PDX model, after 6 weeks of treatment. (*n* = 4–13 xenografts/group, the precise number of xenografts for each PDX is available in the source data file). PD: progressive disease, SD: stable disease, PR: partial response, CR: complete response. **b** Tumour response to cisplatin in the HBCx-9 (complete response), HBCx-11 (partial response), HBCx-151 (stable disease) and the HBCx-39 (progressive disease) PDX. RTV: relative tumour volume. Mean +/− SD. HBCx-9: *n* = 11 and 10 mice in control and treated groups, respectively, HBCx-11: *n* = 11 and 9 mice in control and treated groups, respectively, HBCx-151: *n* = 5 mice/group, HBCx-39: *n* = 11 and 13 mice in control and treated groups, respectively. **c** Response rates according to cisplatin and carboplatin PDX treatment. **d** PDX response to platinum agents according to patients’ neo-adjuvant chemotherapy (AC: Adriamycin and cyclophosphamide; FEC = 5-Fu + epirubicin + cyclophosphamide; NAC: neo-adjuvant chemotherapy). **e** PDX response to platinum agents according to patients’ residual cancer burden (RCB) scores. **f** Kaplan–Meier survival curve with the Gehan–Breslow–Wilcoxon test for the overall survival rate of TNBC patients stratified by platinum response in the matched PDX.

The response of PDX models to platinum (cisplatin or carboplatin) is represented in Fig. 1a. Five (9.1%) and 10 (18.1%) PDX responded with partial (PR) and complete response (CR), respectively, 14 (25.4%) with stable disease (SD) and 26 (47.3%) with progressive disease (PD), according to their best responses and best average responses^15^. Four PDX models are shown as example of complete response (HBCx-9), partial response (HBCx-11), stable disease (HBCx-151) and progressive disease (HBCx-39) in Fig. 1b. PDX were treated by cisplatin or carboplatin without difference in the response rates (53.8% and 50%, respectively) (Fig. 1c). Among the 55 PDX models, 26 (47.3%) were established from primary tumours and 29 (52.7%) from residual tumours after neo-adjuvant chemotherapy (two models were established from patients treated by carboplatin in the neo-adjuvant setting). Sixty-seven percent and 33% of responding models (including both PR and CR) originated from primary tumours and residual tumours, respectively, in contrast to the resistant group (PD) where 35% of PDX originated from primary tumours and 65% from residual tumours (Fig. 1d) (*p* = 0.06, Fisher’s exact test). One patient had a residual cancer burden (RCB) score of 1, 15 patients had a RCB score of 2 and 7 patients had a RCB score of 3. There was a trend towards an increased sensitivity to platinum for PDX established from patients with RCB 1 or 2 (50% of response) as compared to PDX established from patients with RCB 3 (response in only 14% of PDX) (Fig. 1e).

Finally, we compared the overall survival of patients corresponding to platinum responder or resistant PDX. Overall survival was higher in patients corresponding to platinum responder PDX (median survival of 2262 as compared to 579 days for the group of resistant PDX) (Fig. 1f).

In summary, in a large cohort of PDX models, representative of the clinical diversity of early TNBC, platinum-based chemotherapy was effective in more than 50% of tumours. The majority of responding tumours originated from treatment-naïve patients.

### Shallow HRD predicts response to platinum

As *BRCA* mutations and *BRCA1* promoter methylation have been described as potential biomarkers of platinum response^7,8^, we analysed the response in PDX models in relation to these alterations. All PDX were sequenced by targeted DNA sequencing of a panel of 95 genes or 576 genes, as detailed in the methods section. To assess biallelic inactivation of *BRCA1* and *BRCA2* genes, we integrated the loss of heterozygosity (LOH) status of the wild-type allele in the analysis.

**Fig. 2:**
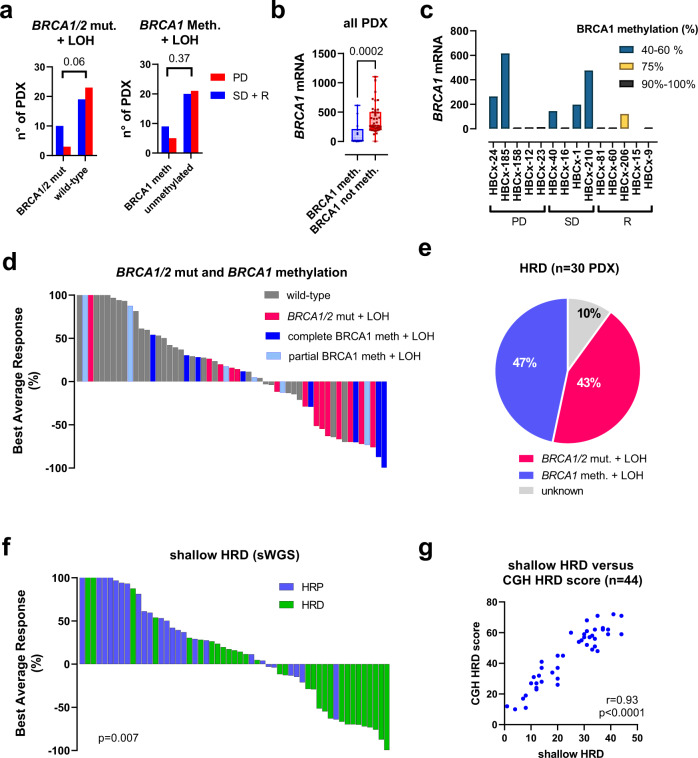
Homologous recombination deficiency in TNBC PDX. **a** Contingency analysis showing the relationship between biallelic *BRCA1/2* inactivation (*BRCA1/2* mutations and *BRCA1* methylation associated with LOH of the second allele) and response to platinum (Fisher’s exact test, two-sided). **b** *BRCA1* gene expression determined by RT-PCR analysis in *BRCA1* methylated PDX (*n* = 14) as compared to un-methylated PDX (*n* = 41). Data are presented as a Min/Max Whiskers plots with lines indicating the median. *p* = 0.0002 (Mann–Whitney test, two-tailed). **c** *BRCA*1 gene expression in *BRCA1* methylated PDX according to the percentage of *BRCA1* methylation. Each bar represents a single value **d** Waterfall plot representing platinum responses in PDX with biallelic inactivation of *BRCA1/2*: pathogenic *BRCA1/2* mutations and *BRCA1* methylation, associated with LOH. **e** Percentage of BRCA1/2 mutation and BRCA1 methylation in the 30 PDX with HRD based on shallow WGS. **f** PDX reponse to platinum according to the HRD status. *p* = 0.007, Fisher’s exact test (two-sided). **g** Correlation analysis between shallowHRD scores and CGH HRD score (Pearson correlation).

Pathogenic mutations in *BRCA1* or *BRCA2* genes associated with LOH of the wild-type allele were present in 13 PDX (23.6%): 10 in the CR + PR + SD group (34.5%) and 3 in the PD group (11.5%) (Fig. 2a). The association between the presence of a *BRCA1/2* mutation and the response to platinum (including SD, CR and PR) was close to statistical significance (*p* = 0.06, Fisher’s exact test). *BRCA1* promoter methylation, an epigenetic mechanism that leads to inhibition of *BRCA1* gene expression, was found in 14 PDX models (25%), 9 in the CR, PR and SD groups and 5 in the PD group (*p* = 0.37, Fisher’s exact test). All *BRCA1* methylated tumours showed LOH of the second *BRCA1* allele. We hypothesized that the lack of association between *BRCA1* methylation and platinum response could depend on incomplete *BRCA1* inactivation in the resistant PDX. Therefore, we analysed the relationship between *BRCA1* methylation, *BRCA1* gene expression and response to platinum. As expected, *BRCA1* gene expression was significantly lower in *BRCA1* methylated PDX as compared to un-methylated models (Fig. 2b). Among *BRCA1* methylated models, however, 5 PDX in the PD and SD groups showed significant residual *BRCA1* gene expression associated with 40–60% of *BRCA1* methylation, while PDX models with no *BRCA1* gene expression exhibited 90-100% of *BRCA1* methylation (Fig. 2c).

There were, however, 3 platinum-resistant PDX with no *BRCA1* gene expression and with complete *BRCA1* methylation, suggesting the existence of additional mechanisms of platinum resistance, beyond incomplete *BRCA1* inactivation.

When analysed together, *BRCA1/2* mutation or *BRCA1* complete methylation predicted response in 51.7% of PDX and lack of *BRCA1/2* mutation or *BRCA1* partial methylation predicted resistance in 76.9% of PDX (*p* = 0.0507, Fisher’s exact test). Thus, a significant proportion of responses (48.3%) are still not predicted by these biomarkers.

We next analysed the response to platinum as a function of the HRD status that was determined from shallow whole-genome sequencing (sWGS), a recently developed procedure for HRD detection based on the number of large-scale genomic alterations^16^. Overall, 30 PDX (54, 5%) were classified as HRD and 25 as non-HRD. Of the HRD, 13 were *BRCA1* or 2 mutated (43.3%), 14 were *BRCA1* methylated (46.7) and 3 were of unknown origin (10%) (Fig. 2d). In the non-HRD group, there were no *BRCA1/2* altered tumours. HRD predicted response (including both CR and PR) in 14/15 (93%), stable disease (SD) in 7/14 PDX (50%), CR + PR + SD in 21/29 PDX (72%). Lack of HRD (Homologous recombination proficiency: HRP) predicted resistance in 17/26 PDX (65%) (Fig. 2e, f; *p* = 0.007, Fisher’s exact test).

Finally, we compared the shallow HRD with the MyChoice HRD, a FDA-approved test of HR deficiency for ovarian cancer. Starting from the copy number data obtained from Array-based comparative genomic hybridization (aCGH) arrays (available for 44 on the 55 PDX), we calculated My-Choice-like HRD scores (CGH scores) from the sum of loss of heterozygosity (LOH), telomeric allelic imbalance (TAI), and large-scale state transitions (LST) scar signature scores^17,18^. The obtained CGH HRD scores were highly correlated with shallow HRD scores (*r* = 0.93, *p* < 0.0001, Pearson correlation analysis) (Fig. 2f) with 95% concordance in HRD prediction. In this set of 44 PDX, sensitivity and specificity in predicting cisplatin response were similar (76% and 61% for the MyChoice-like HRD and 81% and 65% for the shallow HRD).

In summary, these results show that the shallow HRD is a valuable test to predict cisplatin response and identify incomplete *BRCA1* methylation associated to residual gene expression as potential resistance mechanisms in some platinum-resistant PDX.

### Genomic HRD is associated with a defect in homologous recombination repair of cisplatin-induced DNA damage

Twenty-five TNBC PDX of the previous cohort with additional 7 PDX models, established at the Netherlands Cancer Institute (NKI), were further analysed on a genomic level with their matched patients’ tumours and functionally tested for homologous recombination repair based on RAD51 foci formation^19^.

aCGH analysis of PDX and matched patients’ tumours showed that PDX and primary tumours have similar patterns of copy-number aberrations (CNA) and cluster together in the unsupervised clustering (Supplementary Fig. 1a). The 32 PDX models were also analysed by RNAseq and whole-exome sequencing (WES). All models cluster with the basal PAM50 group based on the three-gene model (SCMGENE) molecular subtype^20^ (Supplementary Fig. 1b). Analysis of genomic aberrations in cancer drivers showed that most aberrations consisted of CNA. The only gene that showed mutations in the majority of the samples was *TP53*, with mutations found in primary and/or PDX tumours in 27 out of 32 models (77% of samples) (Supplementary Fig 1c, Supplementary Data 2). In 80% of cases, *TP53* mutation was also detected in the matched patient’s tumours, sometimes with a lower variant allele frequency, possibly reflecting stromal contamination of patients’ samples.

**Fig. 3:**
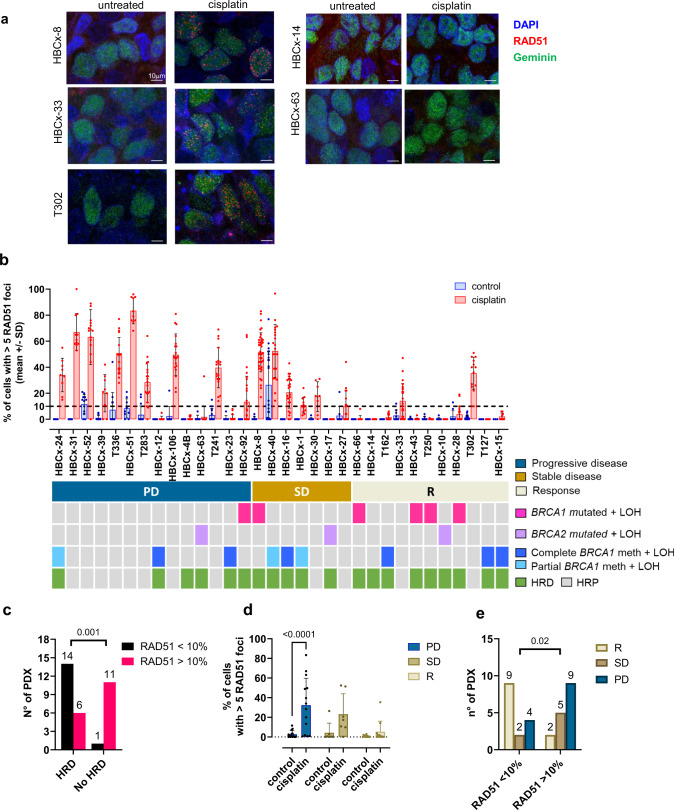
RAD51 formation test in a cohort of PDX models (25 from Institut Curie and 7 from NKI). **a** Representative images of RAD51 staining in untreated and cisplatin-treated xenografts of the PDX HBCx-8, HBCx-33, T302, HBCx-14 and HBCx-63. Red: RAD51, blue: DAPI nuclear stain, green: geminin. Scale bar: 10 μm **b** Percentages of tumour cells with more than 5 RAD51 foci in control and cisplatin-treated xenografts of 32 TNBC PDX. Bar dot plot, mean +/− SD (the n° of mice in each group and PDX is provided in the source data file). **c** Correlation between genomic and functional HRD. *p* = 0.001, Fisher’s exact test, two-sided. **d** Percentages of tumour cells with more than 5 RAD51 foci in control and cisplatin-treated xenografts in PDX from the different response groups (PD, SD, R). Bar dot plot, mean + SD. *n* = 14 (PD), *n* = 7 (SD), *n* = 11 (R). *p* < 0.0001 (PD), Bonferroni’s corrected multiple comparisons test, two-way ANOVA test. **e** N° of PDX with more or less than 10% of RAD51 positive cells in the different response groups. *p* = 0.02, Fisher’s exact test, two-sided.

We next addressed whether PDX with genomic HRD show a defect in repairing cisplatin-induced DNA damage by HR DNA repair, by measuring the RAD51 focus formation in cisplatin-treated xenografts. RAD51 focus formation has been described to predict response to chemotherapy in breast cancer patients^19^. PDX models showing more than 5 RAD51 foci in less than 10% of cells after cisplatin treatment were considered HRD. Immunofluorescence staining of RAD51 foci are shown in Fig. 3a for 3 HR proficient PDX models (HBCx-8, HBCx-33, T302) and for 2 HR deficient PDX models (HBCx-14, HBCx-63). Cisplatin was taken up by tumour cells in these models, as shown by the formation of cisplatin adducts (Supplementary Fig. 2). The amount of RAD51 foci in control and treated xenografts is represented in Fig. 3b and analysed in the context of biallelic inactivation of *BRCA1/2* genes, complete and partial methylation of the *BRCA1* promoter and genomic HRD (sWGS). Fifteen PDX (46.9%) showed 5 or more RAD51 foci in less than 10% of cells: 14 were HRD and 1 HRP based on sWGS, while 17 PDX showed a RAD51 score greater than 10%: 11 were HRP and 6 HRD (Fig. 3b). Functional and genomic HRD were significantly correlated (*p* = 0.001), although 6 HRD PDX were found to be RAD51 proficient, 2 in PD group and 4 in the SD group (Fig. 3b, c). There is a decreasing trend in the proportion of RAD51 positive models in treated samples of the 3 response groups (Fig. 3d) and a low RAD51 score was significantly associated with response, although it failed to predict stable disease that was associated with a high RAD51 score in 5/7 cases (Fig. 3e). Finally, these results show that in the 3 PDX models with partial *BRCA1* methylation (HBCx-24, HBCx-40, HBCx-1) (Fig. 2d) cisplatin treatment results in increased levels of RAD51 foci (Fig. 3b), further supporting the hypothesis that these tumours are not deficient in HR repair, in spite of the genomic scar of HRD and the biallelic inactivation of *BRCA1*. Conversely, 5/6 PDX with complete BRCA1 inactivation do not show any increase of RAD51 levels upon cisplatin treatment and thus can be considered as HR deficient.

### XRCC3 and ORC1 aberrations in cisplatin-sensitive models

**Fig. 4:**
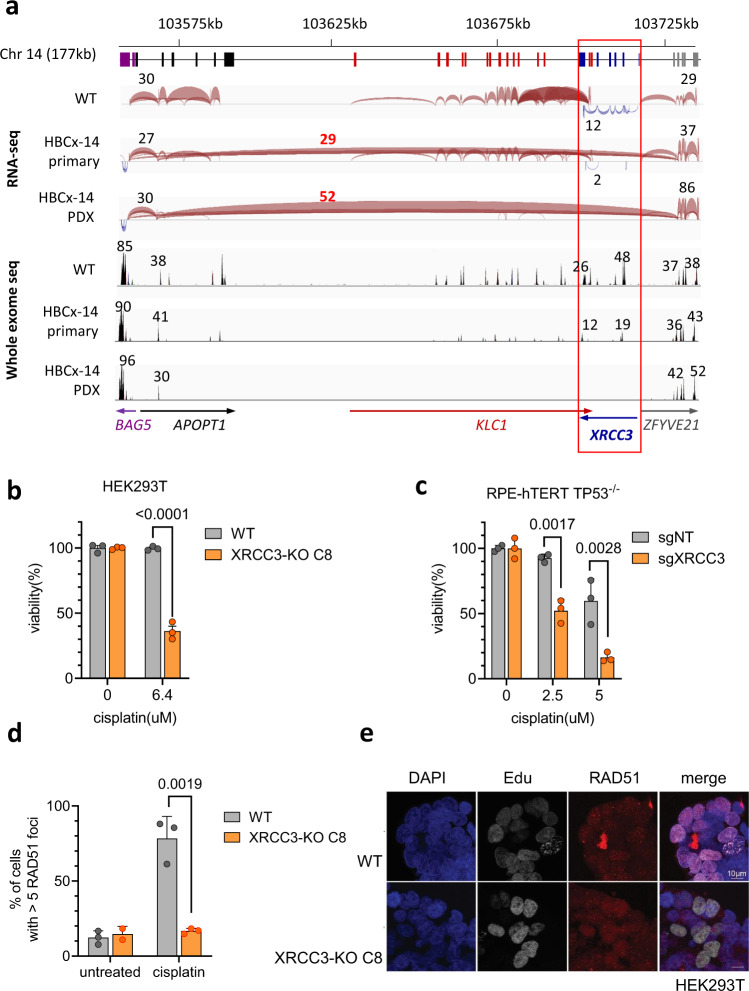
Analysis of cisplatin response in *XRCC3* knockout cells. **a** Schematic overview of a large genomic deletion of a region including *XRCC3* in HBCx-14. Coloured boxes below indicate exons of genes, with gene names and transcriptional direction (arrows) shown in the bottom of the figure. The 3 panels below show the splice junction tracts in IGV for WT (T250 primary), HBCx-14 primary and HBCx-14 xenograft. Splice junctions connecting exons are represented by blue and red arcs, with transcriptional direction of genes indicated by colour. HBCx-14 primary and PDX tumours show a junction from APOPT1 exon 2 to ZFYVE21 exon 2 that is not found in the WT sample. Numbers above and below arcs indicating the junction depth show loss of coverage in the region spanned by the APOPT1-ZFYVE21 junction in HBCx-14 compared to WT, with similar or higher junction coverage outside this region. IGV coverage tracks for WES also shows similar read depth (indicated by numbers above the peaks) outside the region between APOPT1 exon 2 to ZFYVE21 exon 2, with little to no reads inside that region in HBCx-14 tumours. **b** Clonogenic survival assay in HEK293T cells left wild-type (WT) or carrying a frameshifting mutation in *XRCC3*. Value of each data point in survival graph is calculated as a percentage of average absorbance of untreated cells. Survival graph shows a representative of three independent experiments, mean ± SEM. *p* < 0.0001, two-tailed *t*-test. **c** Clonogenic assay in RPE-hTERT p53 KO cells transduced with a non-targeting guide RNA or a guide RNA targeting XRCC3. One representative experiment out of three biological replicates. Mean +/− SEM. *p* = 0.0017 (2.5 μM) and *p* = 0.0028 (5 μM), a two-tailed *t*-test. **d** Quantification of RAD51 foci in HEK293T cells. Mean +/− SD. 3 independent experiments are depicted. *p* = 0.0019, two-tailed *t*-test. **e** Representative images of RAD51 foci after cisplatin treatment for HEK293T WT and the XRCC3 knockout clones. Images show untreated cells and cells treated with 10 µM cisplatin for 3 h, then stained for formation of RAD51 foci and geminin. RAD51 foci are shown in red, Geminin is shown in green, DAPI is shown in blue.

In the cisplatin-sensitive group of this second PDX panel, the sensitivity of three models could not be explained by a mutation or promoter methylation of *BRCA1/2* or mutation or by a mutation in other HR-related genes: HBCx-33, HBCx-14 and T302. We therefore used RNAseq and WES to identify genomic variants that could influence cisplatin sensitivity in these models (Supplementary Fig. 3). Using RNA sequencing we identified fusion transcripts in regions containing DNA damage repair genes (Supplementary Data 3). In HBCx-14 primary and PDX tumours, we found a fusion transcript pointing towards the presence of a genomic deletion on chromosome 14 that encompasses the DNA damage repair gene *XRCC3* (Fig. 4a). The deletion was confirmed in exome sequencing by very low to no coverage of this region in HBCx-14 tumours (Supplementary Fig. 4a).

To investigate the effect of *XRCC3* loss on cisplatin response we used CRISPR/Cas9 to generate *XRCC3* knockouts cells (Supplementary Fig. 4b). As suggested by our analysis, HEK293T cells as well as RPE-hTERT *TP53*^-/-^ cells transduced with guide RNAs targeting *XRCC3* showed increased sensitivity to cisplatin in a clonogenic survival assays when compared to wild-type controls (Fig. 4b, c, Supplementary Fig. 4b–d). Furthermore, these cells were unable to recruit RAD51 to sites of DNA damage after cisplatin treatment (Fig. 4d, e), which is in line with the data obtained from HBCx-14 tumours (Fig. 3a, b).

In the PDX HBCx-33 we identified a point mutation in the DDR gene *TONSL* (c.C3240A, p.N1080K) (Supplementary Data 2). Interestingly, TONSL has been previously reported to promote RAD51 loading at double-strand breaks and stalled replication forks and its depletion confer hypersensitivity to DNA damaging agents and reduced RAD51 foci^21–23^. The N1080K mutation is located within the region required for RAD51 focus formation^23^. Due to the fact that complete loss of *TONSL* was lethal in the cell lines tested, we were unable to test the significance of this mutation with respect to cisplatin response.

**Fig. 5:**
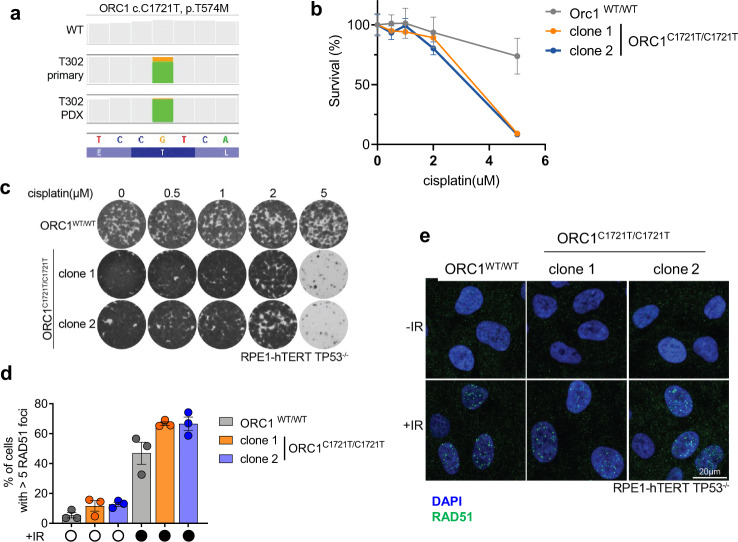
Analysis of cisplatin response in *ORC1* mutated cells. **a** IGV coverage track images showing the identified point mutations in *ORC1* (c.C1721T, p.T574M). Orange/green bar shows the proportion of reads that shows reference base G (orange, bottom panel) compared to mutant base A (green) in T302 primary and PDX tumours. Grey bars show 100% of sequenced bases at that location as reference. Bottom dark/light blue lane shows aminoacids based on reference sequence. **b** Clonogenic survival in untreated and cisplatin-treated WT RPE hTERT TP53^-/-^ cells and cells with a point mutations in *ORC1* (C1721T) introduced using the CRISPR/Cas9 system. Graph shows representative results for three independent experiments. Values of each data point are normalized to untreated conditions of the same genotype and data are depicted as mean ± SEM. **c** Representative images of clonogenic survival after cisplatin treatment for RPE-hTERT TP53^-/-^ cells non-targeted or carrying *ORC1*^*C1721T/C1721T*^. Images show cells left untreated and cells treated with 0.5, 1, 2 or 5 µM cisplatin for 11 days, stained with crystal violet. **d** Formation of RAD51 foci in untreated and cisplatin-treated RPE hTERT TP53^-/-^ cells and cells with CRISPR/Cas9 induced point mutations in *ORC1*. Plot depicts the mean±SEM of RAD51 foci of three independent experiments. Significance was calculated using a two-tailed t-test. **e** Representative images of RAD51 focus formation after irradiation in wild-type cells or *ORC1*^*C1721T/C1721T*^ cells. Cells were irradiated with 10 Grey, then stained for RAD51 foci after 3 h. Rad51 foci are depicted in green, DAPI in blue.

The T302 model showed a mutation in the origin recognition complex gene *ORC1* (c.C1721T, p.T574M) (Fig. 5a, Supplementary Data 2). To investigate if this variant could explains the cisplatin sensitivity of T302 tumours, we used CRISPR/Cas9 to generate RPE-h-TERT *TP53*^*-/-*^ cells with a homozygous C1721T mutations (Supplementary Fig. 5) and found that these cells are indeed more sensitive to cisplatin treatment than WT cells (Fig. 5b, c). As observed in T302 tumours, *ORC1*^*C1721T/C1721T*^ cells still showed RAD51 focus formation after cisplatin treatment (Fig. 5d, e). Similar results were found with an HEK293T cell line containing an *ORC1* deletion (Supplementary Fig. 6).

Finally, to estimate the prevalence of *XRCC3* and *ORC1* alterations in cancer, we queried the cBioPortal database. In the TCGA PanCancer Atlas, homozygous deletions of *XRCC3* were present in different types of cancers, with a low frequency (between 0.2 and 2.8%) (Supplementary Fig. 7a). In breast cancer, *XRCC3* deletions were present in 0.3% of primary tumours (TCGA dataset) and in 2.1% of metastatic breast cancers (Supplementary Fig. 7b). *ORC1* missense mutations were present in the TCGA PanCancer Atlas with a variable frequency (between 0.2 and 5.5%), the tumour types with the highest frequencies were endometrial cancers and cutaneous melanoma (Supplementary Fig. 8a). In breast cancer, *ORC1* mutations were found in 1% of samples (Supplementary Fig. 8b).

## Discussion

In this paper, we studied the response to platinum-based chemotherapy in a large cohort of TNBC PDX models, analysed the predictive value of genomic and functional biomarkers of HRD and identified potential drivers of platinum sensitivity.

Overall, the frequency of cisplatin response including both R and SD, was 54%, which is in the range of responses to platinum-based chemotherapy in early-stage TNBC^24^. At the genomic level, the association of *BRCA1/2* mutations with platinum response was close to significance. A positive correlation between the presence of *BRCA1/2* mutations and platinum-based therapy was found in several studies both in the neo-adjuvant and metastatic setting^7,25–27^, although not in others^28,29^. We find no correlation between cisplatin response and *BRCA1* methylation, in line with recent studies^25,30,31^. Different platinum-resistant *BRCA1* methylated models show significant *BRCA1* gene expression, associated with a low percentage of *BRCA1* methylation and increased levels of RAD51 foci upon cisplatin treatment, supporting incomplete promoter methylation as potential mechanism of platinum resistance in these tumours. This is in accordance with recent studies demonstrating that biallelic *BRCA1/2* inactivation was required for response to PARP inhibitors and platinum^31–33^.

In our study, shallow HRD was predictive of platinum response. Genomic signatures of HRD have been evaluated for their value in predicting response to platinum-based chemotherapy in breast cancer, in both early and advanced breast cancers. The myChoice HRD test predicted response to neo-adjuvant platinum-based chemotherapy in early-stage breast cancers in different clinical trials^17,34,35^. Conversely, in metastatic TNBC, HRD score was not associated with response to platinum-based first line chemotherapy in the TNT trial^30^, but was associated to a better response to platinum-based chemotherapy in patients without *BRCA1/2* germline mutations in the TBCR009 trial^25^.

As sWGS has been shown to robustly detect CNA in formalin-fixed tumours and liquid biopsies of different tumour types, including breast, ovarian and prostate cancer^36,37^, the shallow HRD method is potentially applicable to patients’ samples. Shallow HRD displays efficiency in detecting HRD comparable with the MyChoice HRD and with other tests such as HRDdetect and scarHRD^16^. HRD scores derived from WGS have been shown to reflect those obtained by SNP arrays also by other groups^38^. Limitations of sWGS are related to ploidy estimation and recognition quality of sequencing that can be problematic in some tumours and may introduce a degree of uncertainty^16,38^. For these cases (mostly borderline cases) HRD scores were manually adjusted based on apparent ploidy and recognition quality. We suggest to combine the shallow HRD with other source of genomic information for the classification of tumours with borderline HRD scores close to the threshold. An automated version of our method optimized for clinical diagnostics, with a better classification of the borderline cases without the need of the manual correction, is currently under development.

ShallowHRD was correlated with a lack of functional HR repair, assessed by the formation of RAD51 foci after cisplatin treatment. Lack of RAD51 focus formation has been shown to predict response to neoadjuvant anthracycline-based therapy^19^ or the PARP inhibitor olaparib^39^. Low efficiency in RAD51 focus formation predicted the response in PDX models showing tumour regression and complete response, while the models responding with a stable disease display a high RAD51 score. In the group of resistant PDX, 4 PDX models were RAD51 deficient and HRD. These models, for which both genomic and functional HRD failed to predict resistance, could have developed resistance through a mechanism independent of HR and RAD51 formation restoration, such as increased replication fork protection which has been described for BRCA1/2 deficient cells and has been associated with poor patient response^40–42^.

In 3 PDX models from the responder group without *BRCA1/2* mutations or *BRCA1* promoter methylation, we identified mutations in the DDR genes *XRCC3* (HBCx-14)*, TONSL* (HBCx-33) and *ORC1 (T302)*, that were present also in the matched patients’ primary tumours. By introducing these mutations in cell lines using CRISPR/Cas9, we could demonstrate that mutations in *XRCC3* or *ORC1* genes alter the cisplatin response.

Deleterious variants in *XRCC3*, a RAD51 paralog involved in HR, have been detected in breast and ovarian cancer at a low frequency (0.2%)^43^ and have recently been reported to be mutually exclusive with *BRCA1* and *BRCA2* mutations when analysing data from ovarian, breast, pancreatic and prostate tumours^44^. In cell lines *XRCC3* loss sensitizes cells to IR, crosslinking agents and PARP inhibitors^45,46^ and reduces homology mediated repair^47,48^. While most studies report loss of RAD51 focus formation after *XRCC3* depletion^45,49^, some studies did not observe any impact of *XRCC3* on RAD51 foci formation^47^. Our in vitro experiments do show a role for loss of *XRCC3* in cisplatin sensitivity and loss of RAD51 foci formation in this PDX.

The T574M mutation in the origin recognition complex component *ORC1* has been described in the Meier-Gorlin syndrome, a form of primordial dwarfism as have mutations in other genes from the pre-replication complex^50^. The T574 amino acid is conserved across species and is part of a 20 amino acid long motif involved in the association with chromosomes^51^. Consistent with the role of *ORC1* in the licensing of origins of replication^52^, *ORC1* deficient cell lines show diminished licensing capacity^50,53^. Since normal replication only requires a small percentage of origins to be fired^52^, these cells still are able to proliferate^50,53^. However, when cells encounter replication stress, firing of excess (dormant) origins is required to prevent under-replication of DNA between stalled replication forks^54,55^. Cells with reduced dormant origins are therefore extremely sensitive to DNA damaging agents^56,57^ while still capable of forming RAD51 foci^58^, which is in line with the RAD51 positive phenotype of this cisplatin-sensitive tumour (PDX T302).

In summary, we provide evidence that genomic HRD is highly predictive of response to platinum in a large panel of TNBC PDX. We found residual *BRCA1* gene expression associated to incomplete (mono-allelic) promoter methylation in different HRD tumours in the resistant and stable disease groups. Moreover, we were able to identify a number of genes in addition to *BRCA1/2* that lead to cisplatin sensitivity and confirmed that most tumours sensitive to cisplatin have DNA repair defects. Finally, we also found one cisplatin-sensitive tumour with a mutation that could lead to diminished origin licensing, suggesting there may be tumours that benefit from additional therapeutic approaches.

## Methods

### Ethics statement

Human breast tumour fragments were obtained with patients’ informed consent (Institute Curie) or approval of the Translational Research Board (Netherlands Cancer Institute).

In vivo experimental procedures were approved by the Institutional Animal Care and French Committee (project authorization no. 02163.02) and Netherlands Cancer Institute animal experiments committee and were performed according to respective institutional regulations^5^.

### Patient-derived xenografts and in vivo preclinical assays

To establish PDX models, tumour fragments were removed during surgery of female breast cancer patients and grafted into the interscapular fat pad of 8- to 12-week-old female Swiss nude mice under anaesthesia, as previously detailed^12,59^.

Control and cisplatin/carboplatin treated groups included between 4 and 13 mice, with the exception of HBCx-157 for which 3 xenografts were treated.

When tumours reached a volume of 60 to 200 mm^3^, mice were individually identified and randomly assigned to the control or treated groups, and the treatments were started. Treatment were administered during 6 weeks or less if tumour volumes reach ethical size (2000 mm^3^). Cisplatin (CDDP, Teva) was administered i.p. at 6 mg/kg every 3 weeks (Institut Curie PDX) or by IV injection every 2 weeks (NKI PDX). Carboplatin (Accord) was given by i.p. at 90 mg/kg every 3 weeks. The efficacy of the two schedules of cisplatin administration was compared in one PDX giving similar results (Supplementary Fig. 9). Maximal tumour size/burden was not exceeded.

Tumour growth was evaluated by measurement of two perpendicular diameters of tumours with a caliper twice per week. Individual tumour volumes were calculated as V = *a* × *b*^2^/2, *a* being the largest diameter, *b* the smallest. Tumour volumes were reported to the initial volume as relative tumour volume (RTV). Percent change in tumour volume (ΔVol) was calculated for each tumour as (Vf−V0/V0)*100 where V0 = initial volume (at the beginning of treatment) and Vf = final volume (at the end of treatment).

The best average response was calculated according to Gao^15^. The best response was the minimum value of ΔVol for *t* ≥ 14 d. For each time t and each xenograft, the average of ΔVolt from *t* = 0 to *t* was also calculated. We defined the BestAvgResponse as the minimum value of this average for *t* ≥ 14 d. For each PDX, the median value of best responses and best average responses was calculated. Best response and best average responses were defined as follow:

The criteria for response were defined as follows: complete response (CR), BestResponse <−95% and BestAvgResponse <−40%; partial response (PR), BestResponse <−50% and BestAvgResponse <−20%; stable disease (SD), BestResponse <35% and BestAvgResponse <30%; progressive disease (PD), not otherwise categorized.

### DNA and RNA isolation from fresh-frozen tumour tissue

For isolation of genomic DNA, pieces of snap-frozen tumour tissue were lysed overnight at 55 °C in lysis buffer containing 250 μg/ml proteinase K. DNA was extracted using phenol/chloroform/isoamylalcohol, precipitated with isopropanol and dissolved in TE. Total RNA was isolated from snap-frozen tumour samples using RNA-Bee (Amsbio, UK), using manufacturer’s instructions.

### Shallow whole-genome sequencing and genomic HRD

Genomic DNA was sequenced on HiSeq2500 at a coverage of 1X. FASTQ files were aligned on the hg19 assembly with bwa-mem (v0.7.15)^60^. The mouse reads of the PDX were filtered out using Xenofilter (v1.6)^61^. Duplicate reads and multiple alignments were then filtered out using picard Markduplicates (v2.6; http://broadinstitute.github.io/picard/) and samtools (v1.9)^62^, respectively. Processed bam files were analysed by counting and normalizing the number of reads in fixed window of 50 kb with QDNAseq (v1.20)^63^. To obtain HRD scores, genomic profiles were analysed by shallowHRD (v1.11)^16^. Briefly, initial genomic profile segmentations of QDNAseq were optimized based on minimal CNA cut-off detected for each profile by shallowHRD pipeline. LGAs (large-scale genomic alterations), defined as chromosome arm breaks between adjacent (less than 3 Mb apart) genomic segments of more than 10 Mb, were subsequently called. Samples with more than 20 LGAs were classified as Homologous Recombination Deficient (HRD), while samples with less than 18 LGAs were classified as Homologous Recombination Proficient (HRP). PDX with a borderline HRD score (18 to 20 LGAs) were manually classified as borderline, HRP or HRD, according to apparent ploidy and recognition quality. Two PDX (HBCx-2 and HBCx-95), initially scored with 30 and 21 LGA, were manually corrected to 14 and 20 LGA and classified as HRP, based on false LGA calls and ploidy status (4N).

HRD scores derived from CGH arrays: allele-specific copy numbers were obtained from SNP or cytoscan arrays for 44 PDX using the GAP algorithm^64^. Starting from the copy number data, HRD scores (LOH + LST + TAI) were calculated using a custom R script *(*https://zenodo.org/record/7675801*)*. LOH score was calculated as the number of LOH regions of at least 15 MB but less than the entire chromosome. TAI corresponded to the number of chromosome arms with allelic imbalance at the telomeric side (larger than 500 probes) and allelic balance at the centromere. LST corresponded to the number of allele-specific copy number changes between segments of at least 10 Mb, calculated after filtering alterations less than 3 Mb in size.

### Methylation of *BRCA1*

Methylation of BRCA1 was performed by sodium bisulfite modification of 100 ng of genomic DNA, following the manufacturer’s protocol (EpiTect Plus DNA Bisulfite Kit, Qiagen). The methylated status of the BRCA1 promoter was determined by PCR with specific primers and verified by pyrosequencing (PyroMark Q96 ID Instrument, Qiagen). The degree of CpG methylation was evaluated from the ratios of T and C in the sequence.

### Targeted sequencing of Institut Curie PDX

Institut Curie PDX HBCx-1 to HBCx-181 were analysed by targeted NGS of 95 genes including the most frequently mutated genes in breast cancer (>1%)^12^. PDX HBCx-185 – HBCx-217 were sequenced with a targeted NGS panel (called “DRAGON”) that has been recently developed in the Genetics Department of Institut Curie. It is composed of 576 genes of interest in oncology^65^, including the following genes involved in DNA repair: *ATM, ATR, BARD1, BRCA1, BRCA2, BRIP1, CDK12, CHD1, CHD2, CHD3, CHD4, CHD6, CHD8, CHEK2, ERCC2, FANCA, FANCB, FANCC, FANCD2, FANCE, FANCF, FANCG, FANCI, FANCL, FANCM, NBN, PALB2, RAD21, RAD50, RAD51, RAD51B, RAD51C, RAD51D, RAD54L, RECQL4, SLX4, XRCC2*.

PDX HBCx-4B and HBCx-14 that were found to be HRD but did not show mutations in *BRCA1/2* nor BRCA1 methylation, were sequenced with both panels.

Sequencing for both panels was performed on an Illumina HiSeq2500 with a 500–1000X coverage. Reads were aligned using Burrows-Wheeler Aligner (BWA) allowing up to 4% of mismatches with the reference. Only reads with a mapping quality higher than 20 were used for variant calling, performed with Genome Analysis ToolKit (GATK, v3.5) Unified Genotyper and annotated with COSMIC and 1000 Genome databases^66^. Variants with low allelic frequency (<5%) or low coverage (<100x) and a high 1000 Genome frequency (>0.1%) were excluded from the analysis. The LOH status of the *BRCA1/2* genes was inferred from the variant allele frequencies.

### RAD51 staining

To analyse RAD51 foci, PDX models were treated with a single dose of cisplatin, mice were sacrificed 24 h after treatment and tumour were fixed in formalin. Staining for RAD51 and geminin was done as described previously^19^. Briefly, 3-mm sections of fixed tumours were exposed to antigen retrieval at pH 9 (Dako Target Retrieval Solution, pH 9, Agilent Technologies, reference S2367) for 18 min, then cooled for 20 min and treated by Triton 0.2% for 20 min for permeabilization. Tissue sections were then washed in phosphate-buffered saline (PBS), treated with 100 mL of DNAse I (Roche) for 1 h at 37 °C and blocked with immunofluorescence buffer (IFF; 1% bovine serum albumin, 2% FBS in PBS) for 30 min at room temperature. Sections were stained with geminin antibody in IFF for 1 h at RT, washed with PBS, followed by Alexa Fluor 488 conjugate in IFF for 1 h at RT, washed with PBS, fixed with 4% paraformaldehyde (PFA) solution for 15 min, stained with RAD51 antibody in IFF for 1 h at RT, washed with PBS, followed by anti-mouse Alexa 647 conjugate in IFF for 1 h at RT, washed in PBS with 4’,6-diamidino-2-phenylindole (DAPI; 1:10,000) for 15 min, and fixed again with 4% PFA.

Primary antibodies: anti-RAD51 (mouse, Genetex, GTX70230, Clone 14B4, Lot 44265 (IF 1:250)) and anti-Geminin (rabbit, ProteinTech Group, 10802-1-AP, Lot 00047193 (IF 1:500)). Secondary antibodies: Goat anti-Mouse IgG (H + L) Cross Adsorbed Secondary Antibody, Alexa Fluor 647 (#A-21235, Invitrogen, 1:500) and Goat anti-Rabbit IgG (H + L) Cross Adsorbed Secondary Antibody, Alexa Fluor 488 (#A-11008, Invitrogen, 1:500).

To measure RAD51 foci in HEK293 cells, cells (250000 cells/well) were cultured overnight on coverslips (18 mm) at 37 °C and 5% CO_2_ in DMEM with 10% FCS. Cells were then treated with 10 μM cisplatin for 2 h, then 10 μM EdU was added to the medium and cells were incubated for an additional hour. Medium was removed and cells permeabilized for exactly 1 min in Triton-X100 buffer (0.5% Triton-X100, 20 mM HEPES, 50 mM NaCl, 3 mM MgCl_2_, 300 mM sucrose). Cells were fixed in 4% PFA. EdU staining was performed using the Click-iT kit (Thermofisher). Coverslips were incubated for 30 min in Click-iT reaction cocktail containing Alexa Fluor-488, then washed in 3% PBS/BSA and incubated for 90 min with Rabbit-anti-RAD51 (1:10,000, #2307, a gift from R. Kanaar, Erasmus MC, Rotterdam). After washing, coverslips were incubated with goat-anti-Rabbit Alexa Fluor 594 (1:1000) for 60 min, then mounted in Vectashield mounting medium containing DAPI (Vector Laboratories).

To assess RAD51 foci formation in RPE hTERT p53 KO cells, cells were seeded onto coverslips (VWR), irradiated with 10 Grey of ionizing radiation and left to recover for 3 h. Cells were fixed with 3.7% formaldehyde for 10 min and permeabilized using 5% Triton-X100 diluted in PBS for 10 min. To prevent unspecific binding of the antibody, slides were incubated with blocking solution (1 mg/mL BSA, 3% goat serum, 0.1% Triton-X100, 1 mM EDTA pH 8.0 in PBS) for at least 1 h at room temperature. Subsequently, cells were incubated with the primary RAD51 antibody (Genetex, GTX70230, 1:250) diluted in blocking solution over night at 4 °C. The next day, slides were washed three times with 0.02% Triton-X100 diluted in PBS and incubated with or goat anti-mouse Alexa Fluor-488 (Invitrogen) diluted in blocking solution (1:500) for 1 h at room temperature. Next, slides were again washed with 0.02% Triton-X100 in PBS and mounted in Vectashield mounting medium containing DAPI (Vector Laboratories).

To determine the percentage of RAD51 positive cells, tumour slides or coverslips were imaged on a Leica SP5 confocal system (Leica Microsystems, Germany) equipped with a 63× objective lens. On each slide or coverslip, 3–12 areas were randomly selected and imaged (with a minimum of 100 cells per condition). Foci were counted using ImageJ with an in-house developed macro that measures the number of RAD51 foci for each geminin (for tumour slides), EdU (for HEK293T cells) or DAPI (for RPE-1 cells) positive nucleus. Geminin-positive or EdU-positive cells with more than 5 RAD51 foci were considered positive. For RPE-1 cells, DAPI-positive cells with more than 5 RAD51 foci were considered positive. PDX models showing less than 5 RAD51 foci in less than 10% of cells after cisplatin treatment were considered HR deficient.

### Whole-exome sequencing of paired patients’ and PDX tumour samples

Genomic DNA (1 μg) was fragmented with a Covaris S220 sonicator and DNA fragment libraries were prepared using the TruSeq DNA Sample Preparation Kit (Illumina, Eindhoven, the Netherlands). Library pools (8 libraries/pool) were hybridized to the V4 Exome + UTR kit (Agilent) and sequenced on a Illumina HiSeq (50 bp PE) with a 100X coverage.

Reads were processed and variants filtered as is shown in Supplementary Fig. 3. Reads were trimmed using Cutadapt to remove remaining adaptor sequences, filtering reads shorter than 60 bp after trimming. Trimmed reads were aligned to the human (GRCh38) and mouse (GRCm38) reference genome using BWA. The human alignment was processed for duplicate marking, indel realignment, and base recalibration using Picard Tools and GATK, as recommended by GATK best practices, and filtered to remove contaminating mouse reads using Disambiguate^67^. QC statistics from Fastqc and above-mentioned tools were collected and summarized using Multiqc^68^. Freebayes was used for variant detection. Variants with an alternative depth of less than 2 and an alternative frequency of less than 0.25 were removed. Variants were also removed if they were classified in CLINSIG as benign, were classified as synonymous-SNV, were not exonic or splicing variants, were present in 5 or more of the primary or PDX tumours and/or had a population frequency of more than 0.001 in one of the following databases downloaded with ANNOVAR^69^ (1000 g, Kaviar, hrcr1, gnomad_genome, gnomad_exome, esp6500siv2, exac_03) were excluded. Finally, variants classified as Benign/Tolerated/Possibly damaging/Low/Medium/Neutral in more than 2 of the 5 effect prediction algorithms used (SIFT, Polyphen2_HDIV, MutationAssessor, MetaSVM, FATHMM) were excluded.

The variant list was then filtered for genes included in the Cosmic Cancer gene census list (v84, February 2018; Supplementary Data 4)^70^ to select genes that may act as cancer drivers. To select for genes that may affect the response to cisplatin, variants were filtered against a list of genes implicated in DNA damage response (DDR) (Supplementary Data 5). The gene list for the DDR filtering was compiled by combining genes associated with the following GO-terms: GO:0000077 (DNA damage checkpoint), GO:0000723 (telomere maintenance), GO:0006260 (DNA replication), GO:0006281 (DNA repair), GO:0006301 (Translesion synthesis), GO:0010212 (response to ionizing radiation), GO:0034644 (cellular response to UV), GO:0071478 (cellular response to radiation), GO:0035861 (site of double-strand break).

### Array comparative genomic hybridization (aCGH) analysis

DNA was labelled with Cy3/Cy5 fluorochromes using the Enzo Agilent aCGH labelling kit (Enzo Life Sciences, Raamsdonksveer, the Netherlands) according to manufacturer’s instructions. DNA was hybridized to Nimblegen 12x135K arrays (Roche Nimblegen) according to manufacturer’s instructions, with labelled Human genomic female reference DNA (Promega, Leiden, the Netherlands). Arrays were scanned using an Agilent scanner, and analysed using the Nimblescan software programme.

Logratio’s were transformed into calls with the R-package CGHcall^71^. For the oncoprint, genes with high-level amplifications (call 2) or homozygous deletions (call −2) were selected.

To determine the HRD status of the 7 NKI PDX, CGH profiles were processed using the same shallowHRD pipeline after minor modification of CGH profiles (in each 10 kb window only one randomly selected measured position was retained, others, if any, were discarded).

### RNAseq

Total RNA was analysed using a Agilent Bioanalyzer 2100. Total RNA (100 ng) was used for library generation with the Truseq total RNA library prep kit (Illumina) with Ribozero treatment to remove rRNA. The libraries were sequenced on an Illumina HiSeq2000 using 50-bp paired-end reads. The reads were trimmed using Cutadapt (Martin, 2011)^72^ to remove any remaining adaptor sequences, filtering reads shorter than 20 bp after trimming to ensure good mappability. The trimmed reads were aligned to the GRCh38 reference genome using STAR (version 2.5.2b; ref. ^73^). Mouse reads were filtered out by Disambiguate^67^. QC statistics were from Fastqc^74^ and the above-mentioned tools were collected and summarized using Multiqc^68^. Gene expression counts were generated by featureCounts^75^ using gene definitions from Ensembl GRCh38 version 89. This pipeline is available at (https://github.com/jrderuiter/snakemake-rnaseq).

Normalized expression values were obtained by correcting for differences in sequencing depth between samples using DESeqs median-of-ratios approach (Anders and Huber, 2010)^76^ and then log-transforming the normalized counts. For the TCGA data, normalized gene expression counts were downloaded from Firehose (data set version 2016_01_28) and log-transformed. The PAM50 subtype assignment of the TCGA breast tumours was obtained from the TCGA BRCA publication (Cancer Genome Atlas Network, 2012). Unsupervised clustering (Euclidean distance, average linkage) of the human breast cancer samples from TCGA and the PDX samples was performed using a three-genes signature that distinguishes the PAM50 subtypes^20^.

Fusions were identified with STAR-Fusion^77^ and validated using FusionInspector. To identify deletions, we only included fusion events where fusion partners are located on the same chromosome. Fusions partners and genes in deleted sections between them were filtered against the DDR gene list to select for fusions that include genes that may affect cisplatin sensitivity. To prevent selection of false positives, fusions were only included if the region between fusion points had a summed read count in the lower 10% of normalized read count distribution and all genes in that region had a read count for that specific gene in the lower 10% compared to the rest of the samples.

### Crispr edited *XRCC3* and *ORC1* cell lines

To generate *XRCC3* knockout cells, sgRNAs targeting *XRCC3* (GAACGGCCTCCTTACACTTG) and a non-targeting sgRNA (TGATTGGGGGTCGTTCGCCA) were selected from the GeCKO v2 human gRNA library and were cloned into the pLentCRISPRv2 vector (Addgene, plasmid #52961) as described^78^. All vectors were screened by Sanger sequencing. Lentiviral particles were generated in HEK293T cells using 3rd generation lentiviral vectors and calcium-phosphate transfection. Viruses were titered using a qPCR lentivirus titration kit (Abm, LV900).

HEK293T cells were obtained from ATCC and cultured in Iscove’s medium (Invitrogen Life Technologies) containing 10% FBS, 100 IU/ml penicillin, and 100 μg/ml streptomycin. Transductions were performed by adding diluted viral supernatant to the cells in the presence of 8 μg/mL polybrene (Sigma). Cells were transduced for 24 h with MOI 1, after which cells were refreshed with medium containing 2 μg/mL puromycin. Single colonies were isolated by limiting dilution.

Genomic DNA was isolated using the Gentra Puregene genomic DNA isolation kit (Qiagen). 1 μg of DNA was amplified with specific primers spanning the target site (FW: TATCTGTCCGAGTGCCAGGA; RV: TGTCCACCTCACGCATCTTC) using the Q5 high-fidelity PCR kit (NEB). PCR products were Sanger sequenced using the FW primer and editing efficacy was predicted using TIDE^79^. Untransduced cells were taken along as a control in each sgRNA amplification. Cells were seeded in triplicate at 100,000 cells per well in 6-well plates for clonogenic survival assay. Clonal cell lines were plated in the presence of cisplatin or vehicle, were stained with 0.1% crystal violet 10 days later, scanned with the Gelcount and quantified by extracting crystal violet dye with 10% acetic acid solution crystal violet assay.

Human retinal pigment epithelial cells (RPE-1) were maintained in DMEM GlutaMax (Gibco) supplemented with 10% foetal bovine serum and 1% penicillin streptomycin. RPE-1 cells carrying a p53 deletion and constitutively expressing Cas9^80^ were kindly provided by Daniel Durocher. To generate cells carrying the *ORC1* C1721T point mutation, cells were seeded into 12-well plates the day before and transfected with a guide RNA (GGTCAATGGCATGAAGCTGA) cutting close to the site of the point mutation and a single-stranded DNA template (CAGCCCAAGCCAATGATGTTCCTCCCTTTCAATACATTGAGGTTAACGGCATGAAGCTGATGGAGCCCCACCAAGTCTATGTGCAAATCTTGCAGGTAAGCAGAGCTGTTTAGGCTTTTTG) using Lipofectamine RNAimax (ThermoFisher). Cells were left to recover for 48 h and single-cell clones were generated by limiting dilution. Clones were tested for introduction of the pointmutation by PCR (forward primer: TTATAACGTGTAGTGGCTGG, reverse primer: ATTCTGTCTTCCTTGCCCTT), Sanger sequencing and TIDER analysis^79^.

To assess cisplatin sensitivity of cell lines generated above, 1000 cells/well were seeded in duplicates into 6-well plates and the following day vehicle or cisplatin was added. Colony formation was assessed 9–11 days after seeding depending on confluency. Cell viability was assessed using CellTiterBlue reagent (Promega) according to manufacturer’s instructions. Absorbance was measured on a microplate reader (Infinite200 Pro, Tecan). Percentage of survival was calculated by normalizing values to untreated control cell lines. To stain the cells with crystal violet, cells were fixed with 3.7% formaldehyde and colonies were stained with a 0.1% crystal violet solution.

Cell lines were not authenticated and were regularly tested negative for mycoplasma.

### Statistical analyses

Categorical variables were analysed with the Fisher’s exact test. Sensitivity, specificity, and positive and negative predictive values were calculated with GraphPad Prism software. Two-tailed unpaired t tests were used when comparing two groups. The correlation analysis between shallowHRD and CGH HRD scores was performed by calculating the Pearson correlation coefficient with GraphPad Prism software.

### Reporting summary

Further information on research design is available in the Nature Portfolio Reporting Summary linked to this article.

## Supplementary information

Supplementary Information

Reporting Summary

Supplementary Data 1

Supplementary Data 2

Supplementary Data 3

Supplementary Data 4

Supplementary Data 5

Description of Additional Supplementary Files

## Data Availability

The RNAseq, WES and WGS raw data are available under restricted access due to the possibility of revealing patient-sensitive information. Raw data from RNAseq and WES of Institut Curie and NKI samples have been submitted to The European Genome-phenome Archive (EGA) under the number EGAS00001006393. Request for data access will be referred directly to the Data Access Committee (https://ega-archive.org/dacs/EGAC00001002749) (repository@nki.nl). The WGS raw data have been submitted at EGA under the number EGAS00001005926. Request for data access will be referred directly to the Data Access Committee (https://ega-archive.org/datasets/EGAD00001008839) (data.office@curie.fr). To request access to the datasets, please provide the following information in your Data Access Request: recipient and recipient Institution, details of dataset requested (EGA Study and Dataset Accession Number), brief abstract of the project in which the data will be used, all individuals who will be allowed access to the requested datasets by the recipient institution. After receipt and review of your Data Access Request by the Data Access Committee, you will receive a Data Transfer Agreement to be completed, signed and returned to the Data Access Committee, prior to being granted access to the requested dataset(s). For the avoidance of doubt, the Data Access Committee reserves the right to withhold granting access. The raw data of Institut Curie PDX targeted sequencing are protected due to lack of patients’ consent to deposit in a public repository. Source data are provided with this paper.

## Source data

Source data file

## Code availability

The R script is available at the Zenodo repository (zonodo.org) under the accession number 10.5281/zenodo.7675801.
