# Supplementary material for: Homologous recombination deficiency derived from whole-genome sequencing predicts platinum response in triple-negative breast cancers
Source: Nat Commun. 2023 Apr 7;14:1958. doi: 10.1038/s41467-023-37537-2 (PMC10082194; doi:10.1038/s41467-023-37537-2)

Supplementary Information

Supplementary Figure 1: CGH and RNAseq analysis of TNBC PDX

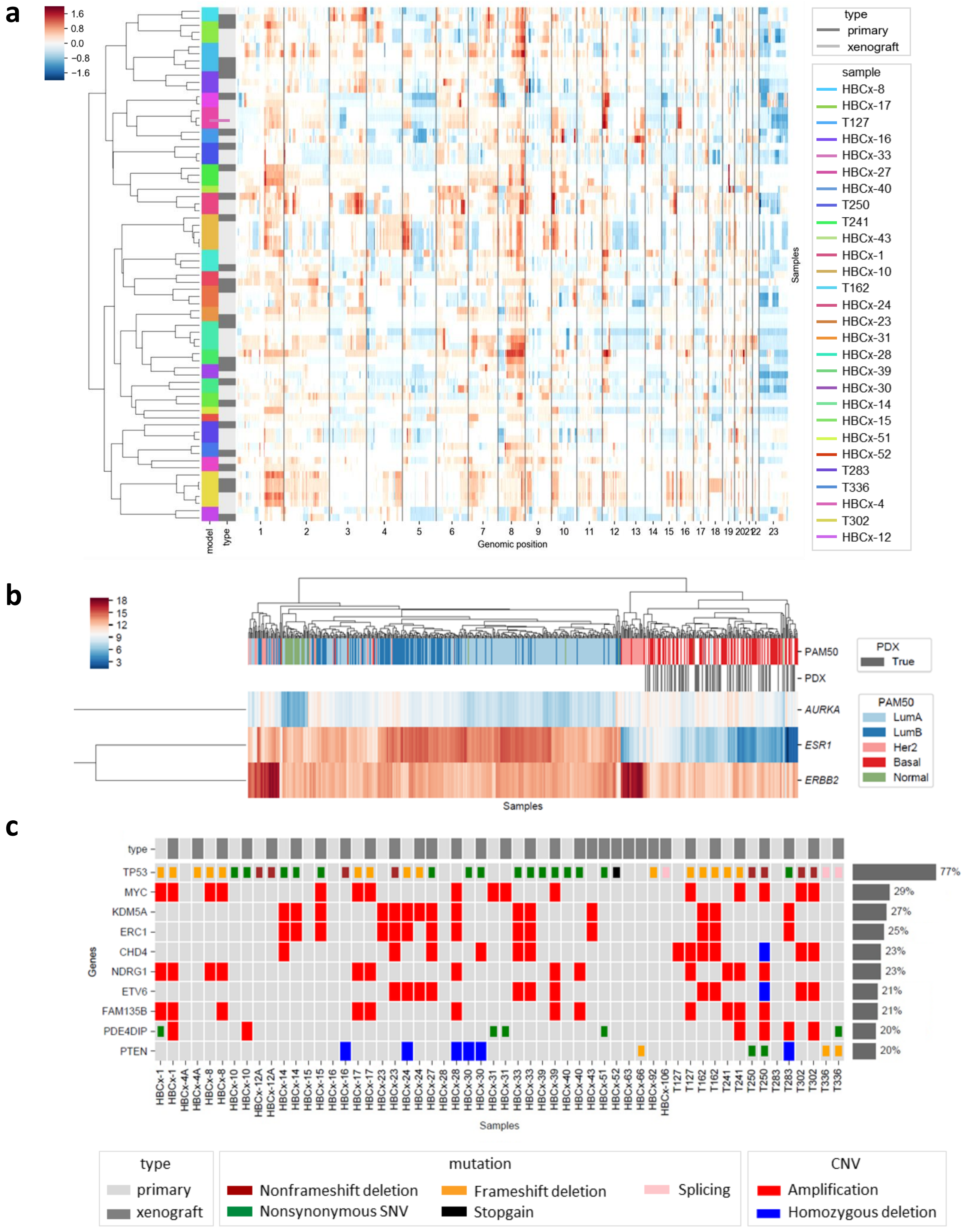

Supplementary Figure 1: Molecular characterization of a panel of triple negative breast PDX models.

a, Unsupervised clustering (correlation distance, average linkage) based on DNA copy number profiles of a panel of 28 TNBC PDX models and corresponding primary tumors.

b, Unsupervised clustering (Euclidean distance, average linkage) of human breast cancer samples from TCGA and from the PDX panel, using a three-genes signature that distinguishes the different PAM50 subtypes. LumA, luminal A; LumB, luminal B; Her2, ERBB2-enriched.

c, Overview of the most frequent mutations and/or copy-number alterations in genes from the Cosmic Cancer gene census (CGC) list in primary and corresponding PDX tumors. Grey bars and percentages on the right indicate the percentage of samples with aberrations in the gene indicated on the left of the figure. CNV = copy number variation.

Supplementary Figure 2: Immunofluorescence staining of cisplatin adducts in control and treated xenografts.

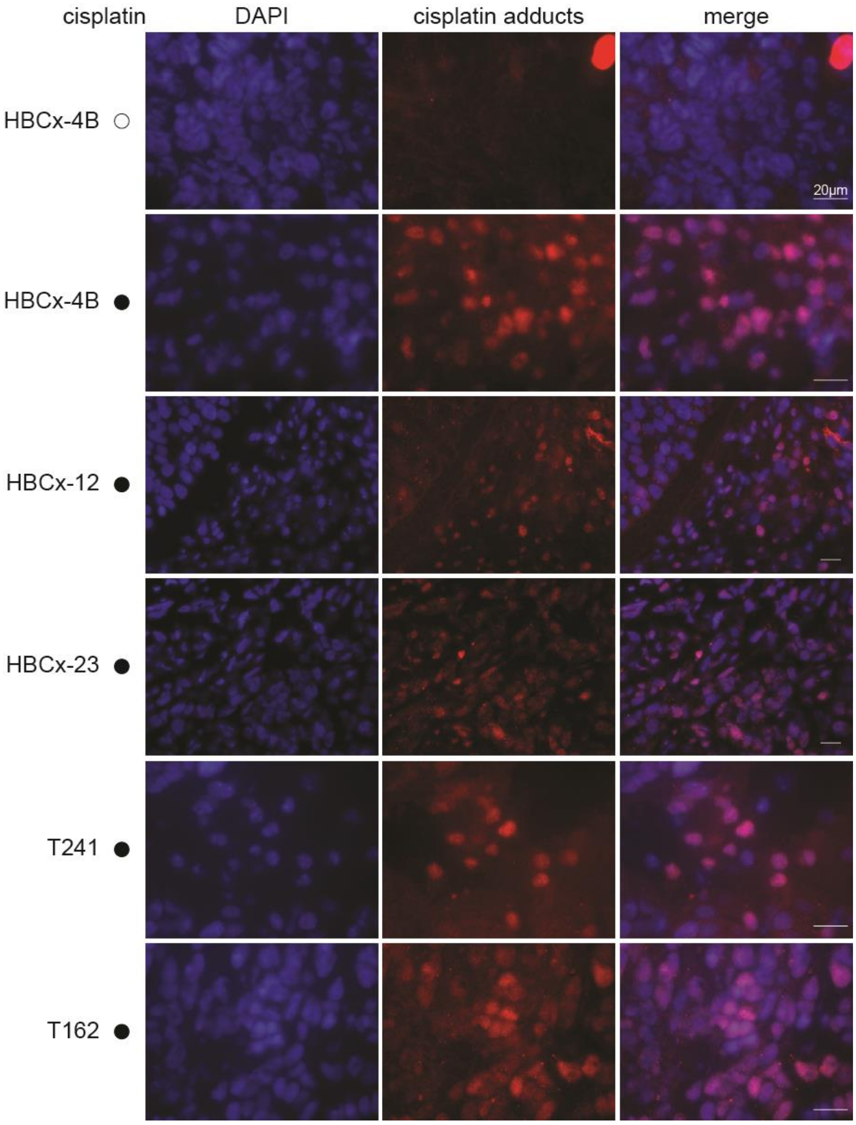

Supplementary Figure 2: Immunofluorescent staining for cisplatin adducts shows presence of cisplatin adducts in tumor cells 24 hours after cisplatin treatment in RAD51 negative, nonresponding models HBCx-4B, HBCx-12B and HBCx-23. The nonresponding RAD51 positive model T241 and the cisplatin sensitive, RAD51 negative model T162 were taken along as controls and show similar patterns of cisplatin staining as the RAD51 negative nonresponding models. White and black circles represent control and cisplatin-treated samples, respectively. Scale bar 20 μm. Representative images of 3 biological replicates are shown.

Supplementary Figure 3: Flowcharts showing pipeline for WES and RNAseq analysis

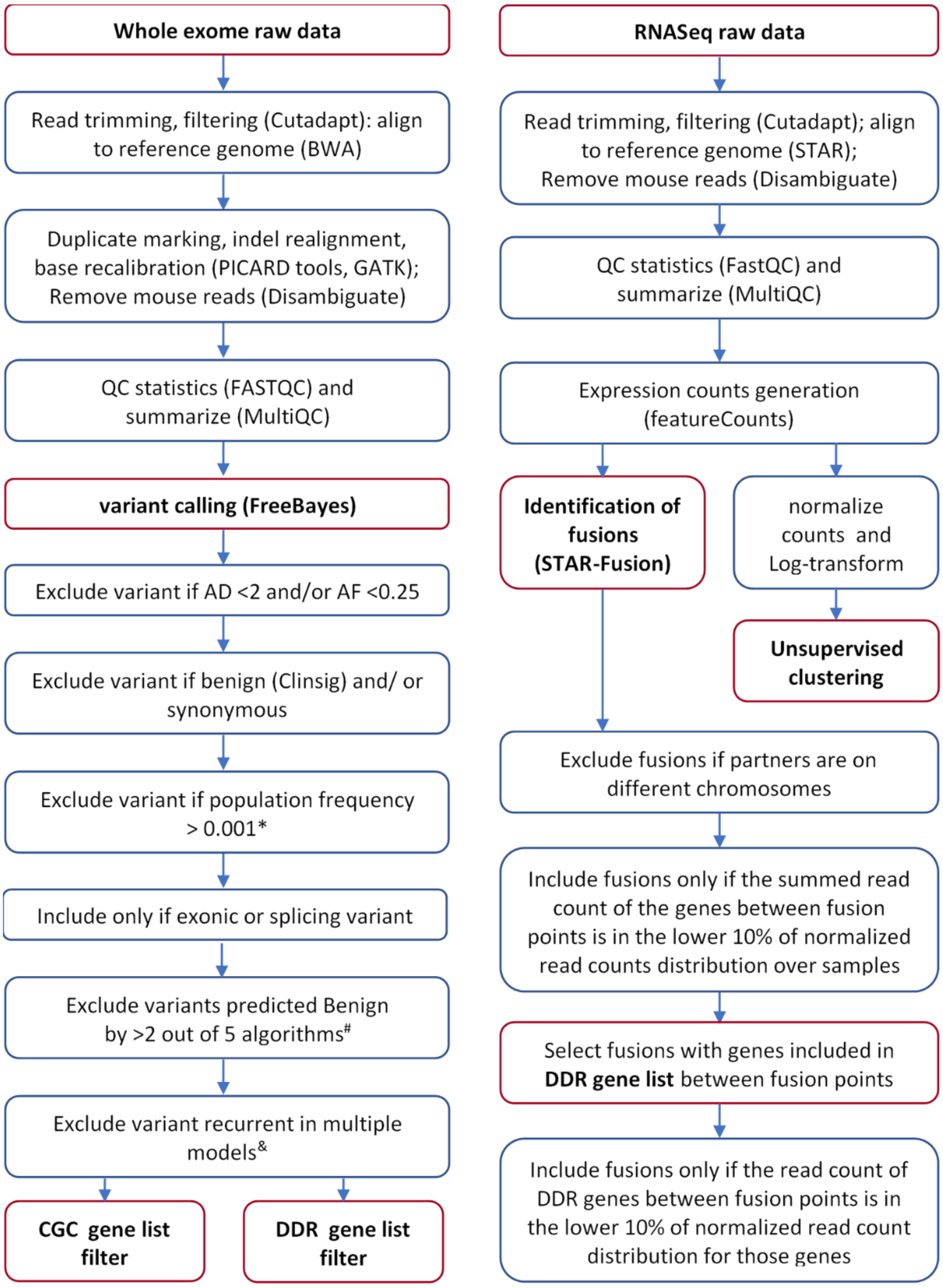

Supplementary Figure 3: Flowcharts showing pipeline for Whole Exome Sequencing analysis (left) and RNA sequencing analysis (right). AD = reading depth for alternative base, AF = frequency of alternative base. * maximum population frequency in 1000g, Kaviar, hrcr1, gnomad_genome, gnomad_exome, esp6500siv2 and exac_03 databases. # prediction algorithms used: SIFT, Polyphen2_HDIV, MutationAssessor, MetaSVM, FATHMM. & excluded if variant is found in 4 of the primary or xenograft tumors. Cancer gene census (CGC) en DNA damage repair (DDR) gene lists are shown in Table S5.

Supplementary Figure 4: XRCC3 KO in HEK293T and RPE cells

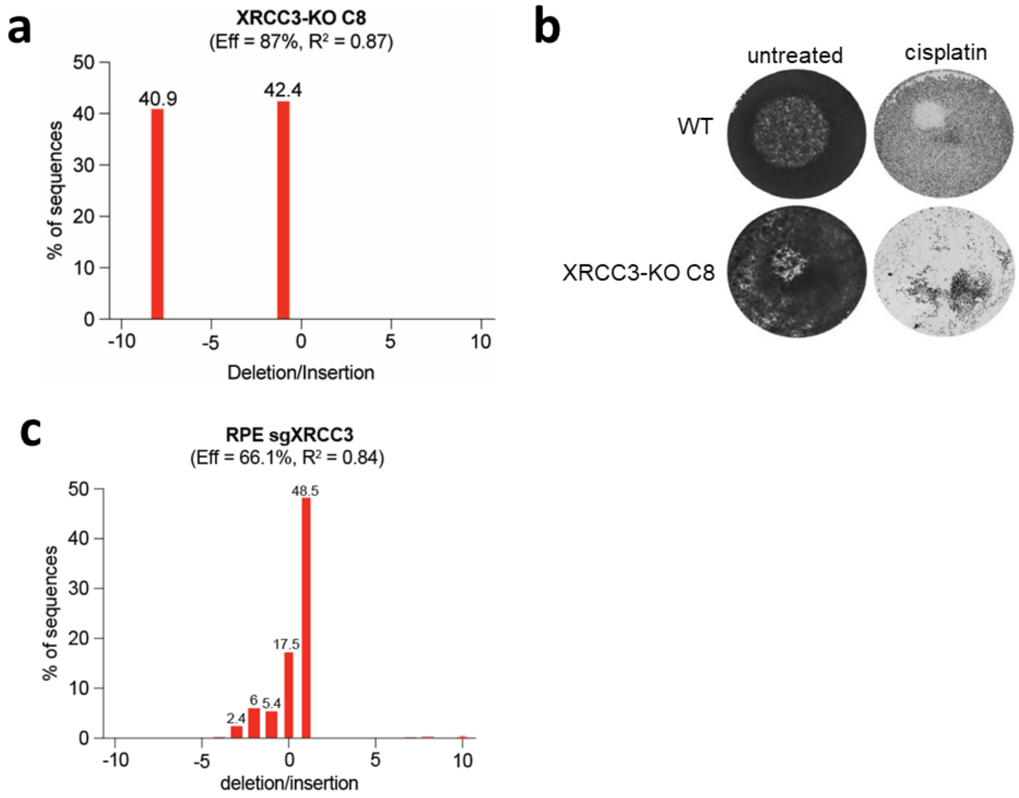

Supplementary Figure 4: XRCC3 KO in HEK293T and RPE cells.

a, TIDE analysis showing the spectrum of insertions/deletions (indels) for the XRCC3 knockout clone 8 in HEK293T cells. Bars show estimated percentages (with number above bars showing percentage) of specific indels, with red bars = $P < 0.001$, two-tailed *t*-test **b,** Colony formation assay in wild type HEK293T cells or XRCC3 KO clone 8. Cells were exposed to 6.4µM of cisplatin and the quantification is depicted in Figure 4B. **c,** TIDE analysis showing the spectrum of insertions/deletion for RPE-hTERT-p53-KO cells transduced with sgXRCC3. Bars show estimated percentages of specific indels ($P<0.001$, two-tailed *t*-test).

Supplementary Figure 5: Validation of ORC1$^{C1721T/C1721T}$ clones in RPE-hTERT cells

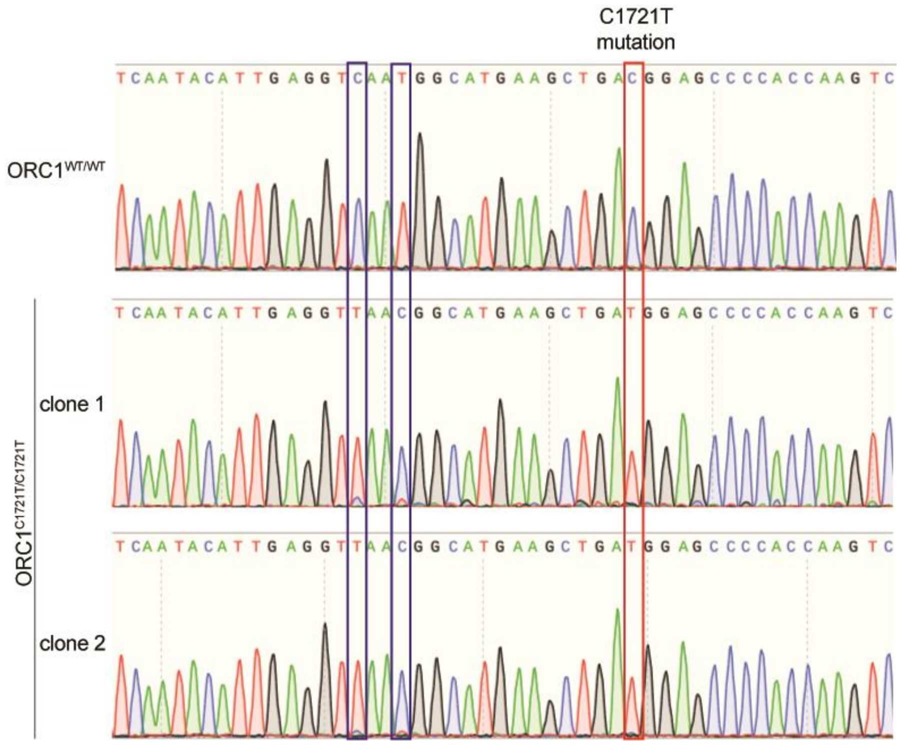

Supplementary Figure 5: **Validation of ORC1$^{C1721T/C1721T}$ clones in RPE-hTERT cells.**

Sanger sequencing tracks of non-edited cells and two individual clones carrying a homozygous C1721T mutation, as indicated by the red box. Additionally two silent mutations have been introduced to prevent re-cutting of the guide RNAs (blue box).

Supplementary Figure 6: Clonogenic survival and RAD51 foci in ORC1 targeted cells

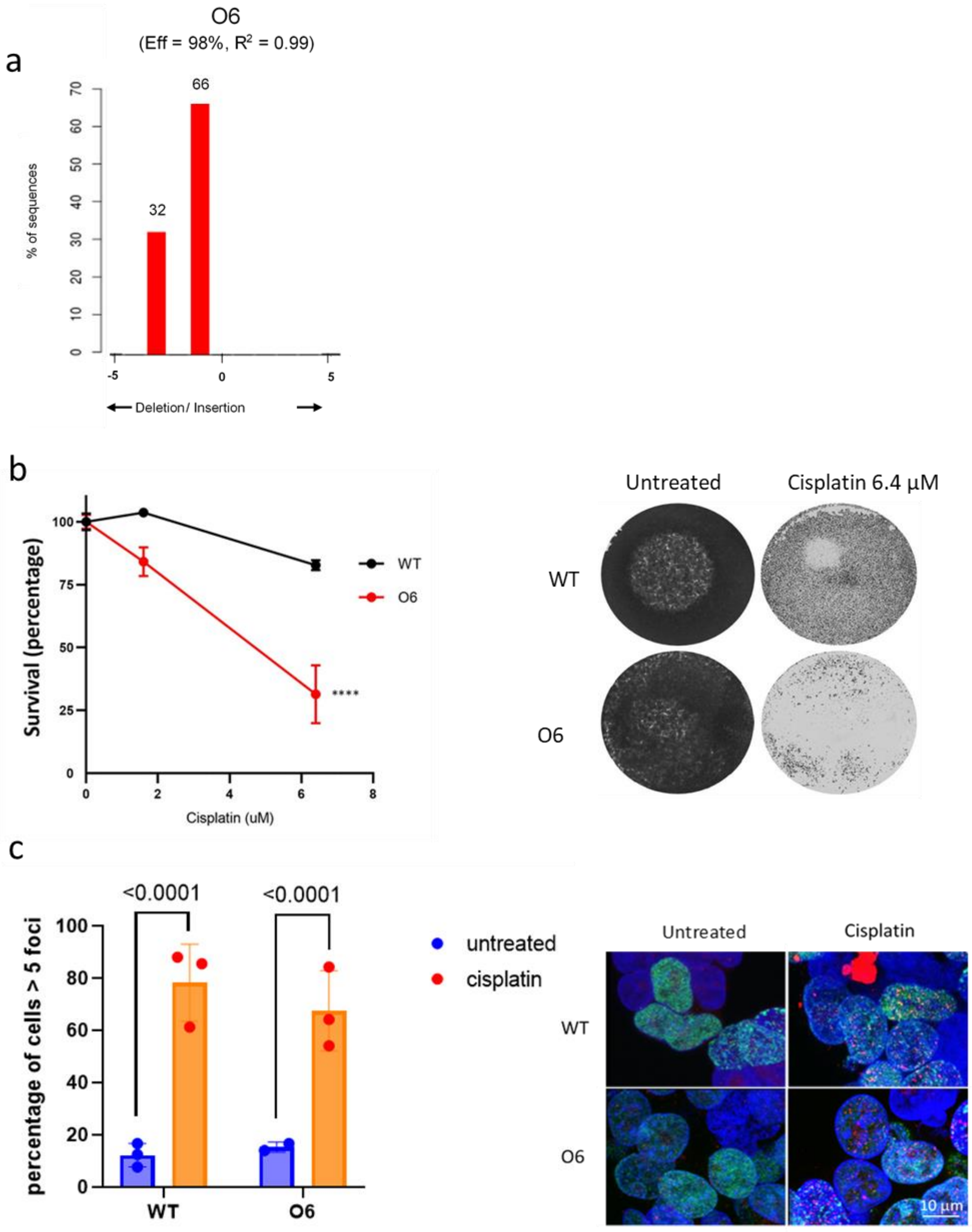

Supplementary Figure 6 : Clonogenic survival and RAD51 foci in ORC1 targeted cells

a, TIDE analysis showing the spectrum of insertions/deletions (indels) for targeted ORC1 clone O6. Bars show estimated percentages (with number above bars showing percentage) of specific indels, with red bars = p < 0.001, two-tailed t-test. Graph shows heterozygous indels, with no WT alleles left. **b,** Clonogenic survival (left panel) and with representative images (right panel)

in untreated and cisplatin treated WT HEK293T cells and cells with CRISPR/Cas9 induced mutations in ORC1 (O6). Value of each data point in survival graph is calculated as percentage of average absorbance of untreated cells. Survival graph shows average +/- SD, Representative graph of 3 biological replicates is shown. *P* values are shown as **** = p<0.0001, two-tailed *t*-test. **c,** Quantification of RAD51 foci (left panel) and representative images (right panel) of RAD51 foci in untreated and cisplatin treated WT HEK293T cells and cells with CRISPR/Cas9 induced mutations in ORC1 (O6). Bars in RAD51 foci graph average +/- SD for 3 independent experiments. Significance was calculated by a two-tailed *t*-test. Graph shows significantly increased percentage of cells with more than 5 foci after cisplatin treatment in both WT and ORC1 mutated cells, with no difference in between cisplatin treated WT and ORC1 mutated cells. Images on the right show RAD51 foci in untreated cells and cells treated with 10 µM cisplatin for 3 hours, then stained for formation of RAD51 foci and geminin. RAD51 foci are shown in red, Geminin is shown in green, DAPI is shown in blue. Scale bar: 10 µm.

Supplementary Figure 7: *XRCC3* alterations in human cancers

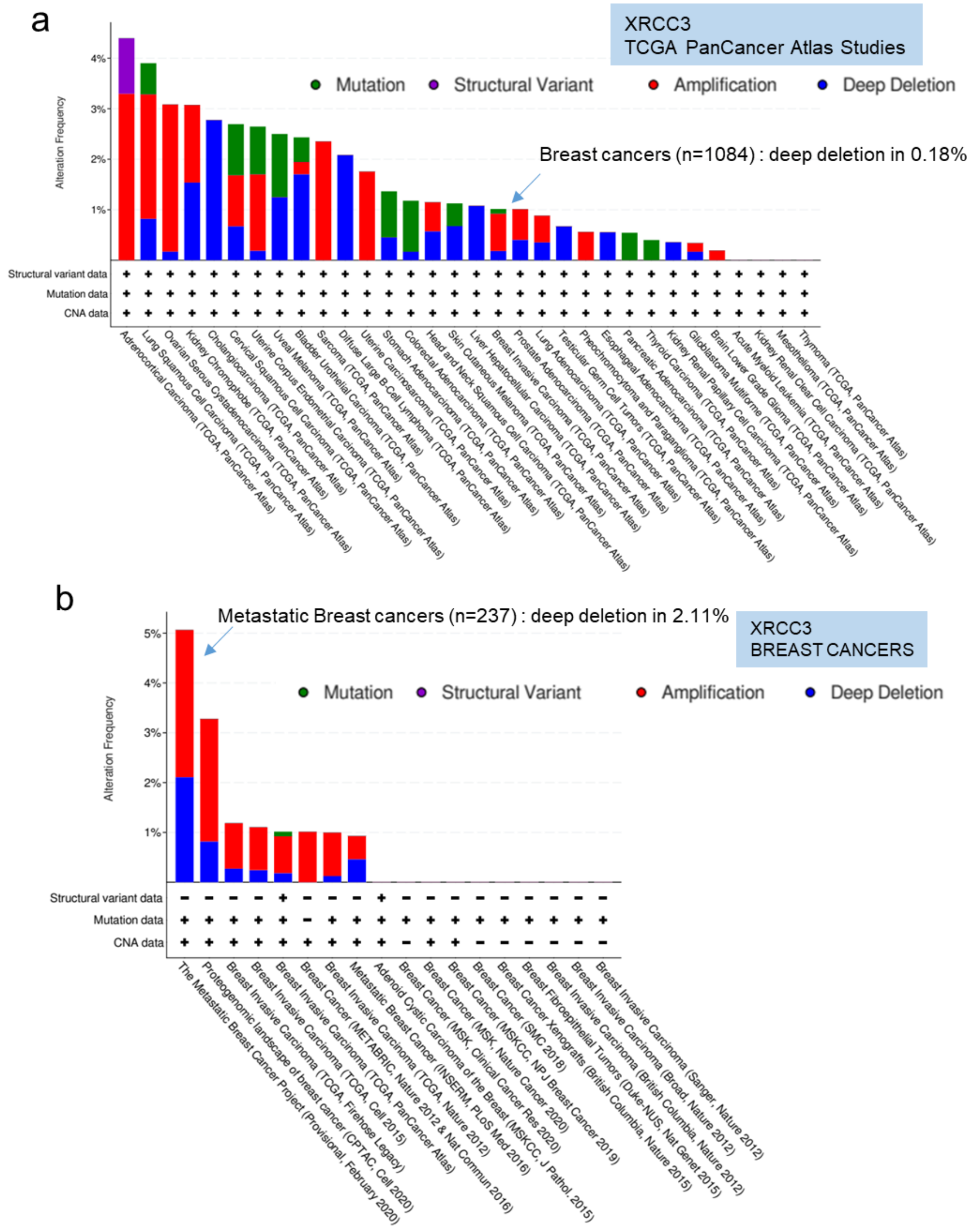

Supplementary Fig. 7: *XRCC3* alterations in human cancers

a, *XRCC3* genomic alterations in patients' tumours from the TCGA PanCancer Atlas Studies.

b, *XRCC3* genomic alterations in breast cancer patients

Supplementary Figure 8: *ORC1* alterations in human cancers

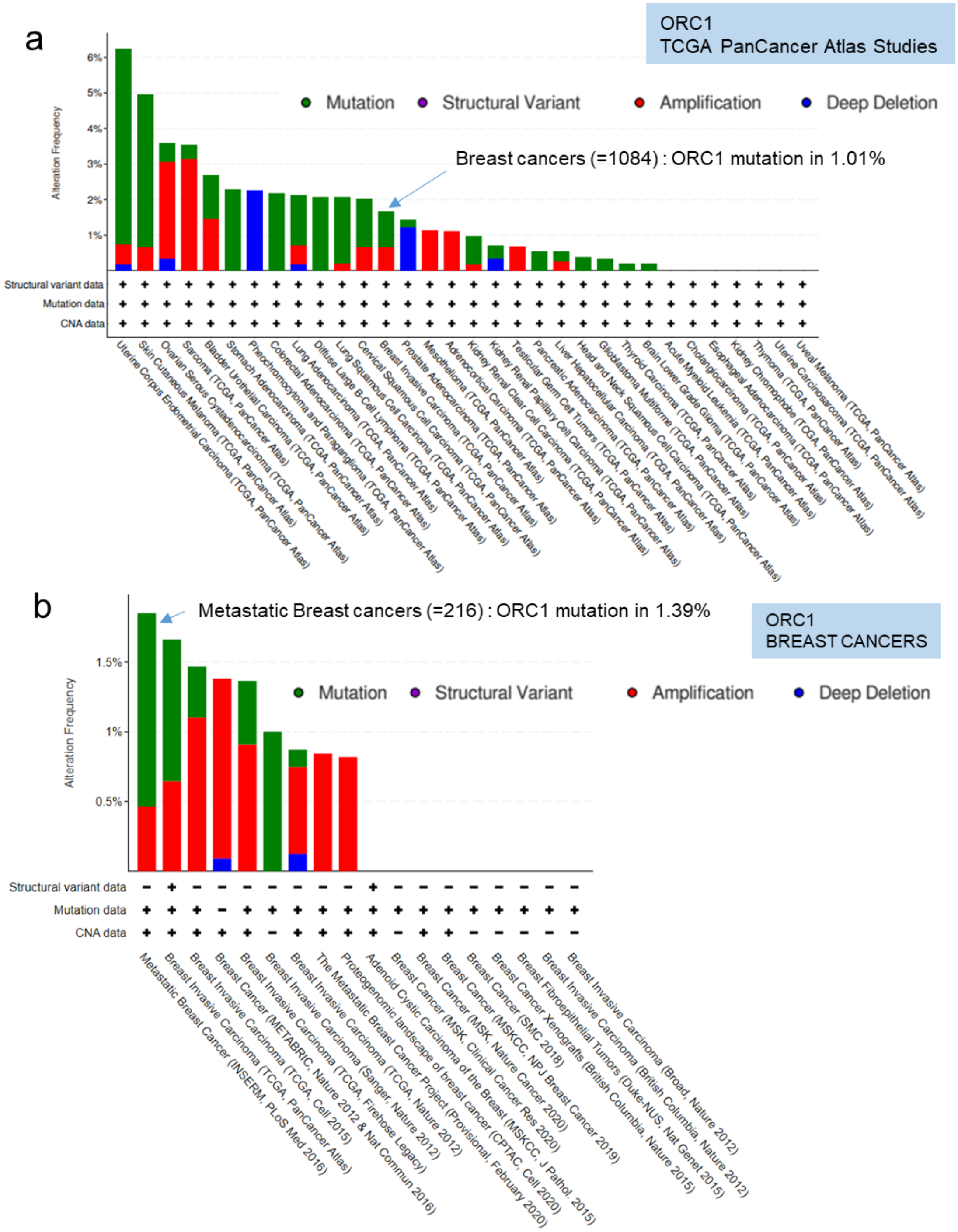

Supplementary Fig. 8: *ORC1* alterations in human cancers

a, *ORC1* genomic alterations in patients' tumours from the TCGA PanCancer Atlas Studies. **b,** *ORC1* genomic alterations in breast cancer patients

Supplementary Figure 9: Response to cisplatin given by IV every 2 weeks and by IP every 3 weeks in the HBCx-17 PDX (individual tumour growth curves).

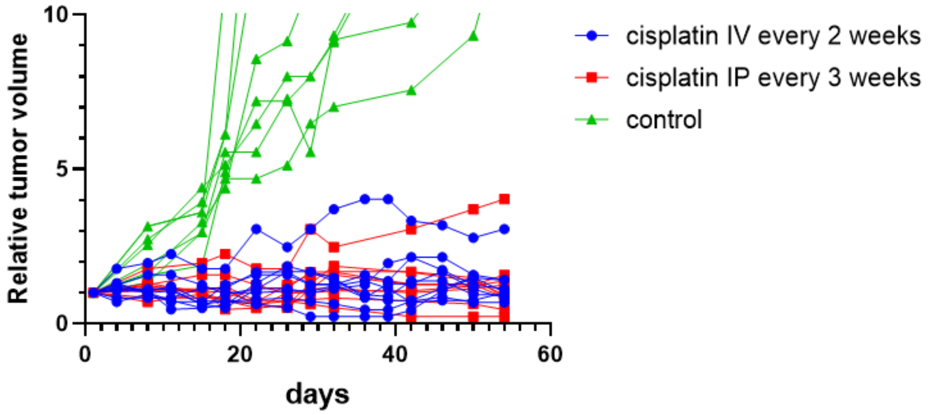

Supplement: Supplementary file 1 — Supplementary Information [file 41467_2023_37537_MOESM1_ESM.pdf]
