# Supplementary material for: Homologous recombination deficiency derived from whole-genome sequencing predicts platinum response in triple-negative breast cancers
Source: Nat Commun. 2023 Apr 7;14:1958. doi: 10.1038/s41467-023-37537-2 (PMC10082194; doi:10.1038/s41467-023-37537-2)
Supplement: Supplementary file 2 — Reporting Summary [file 41467_2023_37537_MOESM2_ESM.pdf]

nature portfolio

Corresponding author(s): ELisabetta Marangoni
NCOMMS-22-14715B

Last updated by author(s): 2023/02/14

Reporting Summary

Nature Portfolio wishes to improve the reproducibility of the work that we publish. This form provides structure for consistency and transparency in reporting. For further information on Nature Portfolio policies, see our Editorial Policies and the Editorial Policy Checklist.

Statistics

For all statistical analyses, confirm that the following items are present in the figure legend, table legend, main text, or Methods section.

n/a	Confirmed	
☐	☒	The exact sample size (*n*) for each experimental group/condition, given as a discrete number and unit of measurement
☒	☐	A statement on whether measurements were taken from distinct samples or whether the same sample was measured repeatedly
☐	☒	The statistical test(s) used AND whether they are one- or two-sided<br>*Only common tests should be described solely by name; describe more complex techniques in the Methods section.*
☒	☐	A description of all covariates tested
☐	☒	A description of any assumptions or corrections, such as tests of normality and adjustment for multiple comparisons
☐	☒	A full description of the statistical parameters including central tendency (e.g. means) or other basic estimates (e.g. regression coefficient) AND variation (e.g. standard deviation) or associated estimates of uncertainty (e.g. confidence intervals)
☐	☒	For null hypothesis testing, the test statistic (e.g. *F*, *t*, *r*) with confidence intervals, effect sizes, degrees of freedom and *P* value noted<br>*Give P values as exact values whenever suitable.*
☒	☐	For Bayesian analysis, information on the choice of priors and Markov chain Monte Carlo settings
☒	☐	For hierarchical and complex designs, identification of the appropriate level for tests and full reporting of outcomes
☐	☒	Estimates of effect sizes (e.g. Cohen's *d*, Pearson's *r*), indicating how they were calculated

Our web collection on statistics for biologists contains articles on many of the points above.

Software and code

Policy information about availability of computer code

Data collection	Tumor sizes of PDX models were collected with a caliper and recorded in Excel (v1997-2003, Microsoft Office). Tumor volumes were calculated in Excel files.<br>Shallow whole genome sequencing (sWGS): FASTQ files were aligned on the hg19 assembly with bwa-mem (v0.7.15). The mouse reads of the PDX were filtered out using Xenofilter (v1.6;) . Duplicate reads and multiple alignments were then filtered out using picard Markduplicates (v2.6; http://broadinstitute.github.io/picard/) and samtools (v1.9), respectively.<br>Targeted sequencing : Sequencing for both panels (95 and 576 genes) were performed on an Illumina HiSeq2500 with a 500–1000X coverage. Reads were aligned using Burrows-Wheeler Aligner (BWA v0.7.17) allowing up to 4% of mismatches with the reference.<br>Whole exome sequencing (WES): Genomic DNA (1 µg) was fragmented with a Covaris S220 sonicator and DNA fragment libraries were prepared using the TruSeq DNA Sample Preparation Kit (Illumina, Eindhoven, the Netherlands). Library pools (8 libraries/ pool) were hybridized to the V4 Exome + UTR kit (Agilent) and sequenced on a Illumina HiSeq (50bp PE).<br>Array Comparative Genomic Hybridization (aCGH) : DNA was labeled with Cy3/ Cy5 fluorochromes using the Enzo Agilent aCGH labelling kit (Enzo Life Sciences, Raamsdonksveer, the Netherlands) according to manufacturer's instructions. DNA was hybridized to Nimblegen 12x135K arrays (Roche Nimblegen) according to manufacturer's instructions, with labeled Human genomic female reference DNA (Promega, Leiden, the Netherlands). Arrays were scanned using an Agilent scanner.<br>RNAseq: Total RNA (100 ng) was used for library generation with the Truseq total RNA library prep kit (Illumina) with Ribozero treatment to remove rRNA. The libraries were sequenced on an Illumina HiSeq2000 using 50-bp paired-end reads. The reads were trimmed using Cutadapt (v.1.13) (Martin, 2011) to remove any remaining adapter sequences, filtering reads shorter than 20 bp after trimming to ensure good mappability.<br>RAD51 staining: to determine the percentage of RAD51 positive cells, tumor slides or coverslips were imaged on a Leica SP5 confocal system (Leica Microsystems, Germany) equipped with a 63x objective lens. On each slide or coverslip, 5-7 areas were randomly selected and imaged (with a minimum of 100 cells per condition).
Data analysis	For the in vivo studies, the best responses and the best average responses were calculated with the Excel software (v1997-2003). The softwrare GraphPad Prism (v9.5.1) was used to represent tumor growth curves and waterfall plots and to perform the statistical analyses.<br><br>Analysis of shallow WGS: Processed bam files were analysed by counting and normalizing the number of reads in fixed window of 50kb with QDNAseq (v1.20) . To obtain HRD scores, genomic profiles were analysed by shallowHRD (v1.11) . Briefly, initial genomic profile segmentations of QDNAseq were optimized based on minimal copy number alteration (CNA) cut-off detected for each profile by shallowHRD pipeline. LGAs (large-scale genomic alterations), defined as chromosome arm breaks between adjacent (less than 3Mb apart) genomic segments of more than 10Mb, were subsequently called. Samples with more than 20 LGAs were classified as Homologous Recombination Deficient (HRD), while samples with less than 18 LGAs were classified as Homologous Recombination Proficient (HRP). PDX with a borderline HRD score (18 to 20 LGAs) were manually classified as borderline, HRP or HRD, according to apparent ploidy, recognition quality and mutational status.<br>cCGH Arrays from NKI were analyzed using the Nimblescan software program (v2.5) .<br>HRD scores derived from CGH arrays : allele-specific copy numbers were obtained from SNP or cytoscan arrays for 44 PDX using the GAP algorithm (PMID: 19903341). Starting from the copy number data, HRD scores (LOH + LST + TAI) were calculated using a custom R script. LOH score was calculated as the number of LOH regions of at least 15 MB but less than the entire chromosome. TAI corresponded to the number of chromosome arms with allelic imbalance at the telomeric side (larger than 500 probes) and allelic balance at the centromere. LST corresponded to the number of allele-specific copy number changes between segments of at least 10Mb, calculated after filtering alterations less than 3Mb in size.<br><br>Analysis of targeted sequencing: Only reads with a mapping quality higher than 20 were used for variant calling, performed with Genome Analysis ToolKit (GATK, v3.5) Unified Genotyper and annotated with COSMIC and 1000 Genome databases . Variants with low allelic frequency (<5%) or low coverage (<100x) and a high 1000 Genome frequency (>0.1%) were excluded from the analysis.<br><br>Analysis of WES: Reads were processed and variants filtered as is shown in supplementary figure 3. Reads were trimmed using Cutadapt (v.1.13) to remove remaining adapter sequences, filtering reads shorter than 60 bp after trimming. Trimmed reads were aligned to the human (GRCh38) and mouse (GRCm38) reference genome using BWA. The human alignment was processed for duplicate marking, indel realignment, and base recalibration using Picard Tools and GATK (v3.5), as recommended by GATK best practices, and filtered to remove contaminating mouse reads using Disambiguate (v. 2016.11.10). QC statistics from Fastqc and above-mentioned tools were collected and summarized using Multiqc (v.1.0). Freebayes (v.1.0.2) was used for variant detection. Variants with an alternative depth of less than 2 and an alternative frequency of less than 0.25 were removed. Variants were also removed if they were classified in CLINSIG as benign, were classified as synonymous-SNV, were not exonic or splicing variants, were present in 5 or more of the primary or PDX tumors and/or had a population frequency of more than 0.001 in one of the following databases downloaded with ANNOVAR 67 (v. 2015_08) (1000g, Kaviar, hrcr1, gnomad_genome, gnomad_exome, esp6500siv2, exac_03) were excluded. Finally, variants classified as Benign/ Tolerated/ Possibly damaging/ Low/ Medium/ Neutral in more than 2 of the 5 effect prediction algorithms used (SIFT, Polyphen2_HDIV, MutationAssessor, MetaSVM, FATHMM) were excluded. The variant list was then filtered for genes included in the Cosmic Cancer gene census list (2018_03).<br><br>aCGH: Logratio's were transformed into calls with the R-package CGHcall (v3.5). For the oncoprint, genes with high-level amplifications (call 2) or homozygous deletions (call -2) were selected.<br><br>RNAseq: The trimmed reads were aligned to the GRCh38 reference genome using STAR (version 2.5.2b). Mouse reads were filtered out by Disambiguate 65 (v. 2016.11.10). QC statistics were from Fastqc 71 and the above-mentioned tools were collected and summarized using Multiqc . Gene expression counts were generated by featureCounts 72 (v. 1.5.2) using gene definitions from Ensembl GRCh38 version. This pipeline is available at (https://github.com/jrderuiter/snakemake-rnaseq). Normalized expression values were obtained by correcting for differences in sequencing depth between samples using DESeqs median-of-ratios approach (Anders and Huber, 2010) and then log-transforming the normalized counts. For the TCGA data, normalized gene expression counts were downloaded from Firehose (data set version 2016_01_28) and log-transformed. The PAM50 subtype assignment of the TCGA breast tumors was obtained from the TCGA BRCA publication (Cancer Genome Atlas Network, 2012). Unsupervised clustering (Euclidean distance, average linkage) of the human breast cancer samples

from TCGA and the PDX samples was performed using a three-genes signature that distinguishes the PAM50 subtypes 19.

RAD51 staining: RAD51 foci were counted using ImageJ (v1.5.0) with an in-house developed macro that measures the number of RAD51 foci for each geminin (for tumor slides), Edu (for HEK293T cells) or DAPI (for RPE-1 cells) positive nucleus. Geminin positive or Edu positive cells with more than 5 RAD51 foci were considered positive. For RPE-1 cells, DAPI positive cells with more than 5 RAD51 foci were considered positive.

For manuscripts utilizing custom algorithms or software that are central to the research but not yet described in published literature, software must be made available to editors and reviewers. We strongly encourage code deposition in a community repository (e.g. GitHub). See the Nature Portfolio guidelines for submitting code & software for further information.

Data

Policy information about availability of data

All manuscripts must include a data availability statement. This statement should provide the following information, where applicable:

- Accession codes, unique identifiers, or web links for publicly available datasets
- A description of any restrictions on data availability
- For clinical datasets or third party data, please ensure that the statement adheres to our policy

The RNAseq, WES and WGS raw data are available under restricted access due to the possibility of revealing patient-sensitive information.
Raw data from RNAseq and WES of Institut Curie and NKI samples have been submitted to The European Genome-phenome Archive (EGA) under the number EGAS00001006393. Request for data access will be referred directly to the Data Access Committee (https://ega-archive.org/dacs/EGAC00001002749) (repository@nki.nl).
The WGS raw data have been submitted at EGA under the number EGAS00001005926. Request for data access will be referred directly to the Data Access Committee (https://ega-archive.org/datasets/EGAD00001008839) (data.office@curie.fr).
To request access to the datasets, please provide the following information in your Data Access Request: recipient and recipient Institution, details of dataset requested (EGA Study and Dataset Accession Number), brief abstract of the project in which the data will be used, all individuals who will be allowed access to the requested datasets by the recipient institution. After receipt and review of your Data Access Request by the Data Access Committee, you will receive a Data Transfer Agreement to be completed, signed and returned to the Data Access Committee, prior to being granted access to the requested dataset(s). For the avoidance of doubt, the Data Access Committee reserves the right to withhold granting access.
The raw data of Institut Curie PDX targeted sequencing are protected due to lack of patients' consent to deposit in a public repository.

Human research participants

Policy information about studies involving human research participants and Sex and Gender in Research.

Reporting on sex and gender	PDX were established from female breast cancer patients. No information/data about gender were collected.
Population characteristics	The PDX used in this study were established from patients with triple-negative breast cancer treated at Institut Curie (n=55) or Natherland Cancer Institute (n=7), with their informed consent.<br>The cohort of 55 patients is described in Table 1 and presented the usual characteristics of TNBC . Mean age at diagnosis was 54 years. TNM staging mostly corresponded to T2 (55%), N0 (65%) with a small percentage of synchronous metastasis (7%). Histologically, 89% of tumours were invasive ductal carcinoma of no special type (NST) and 11% were metaplastic carcinomas. Ninety-five percent of patients had a high SBR histological grade. The majority of patients received sequential chemotherapy (anthracycline then taxane) as adjuvant or neoadjuvant therapy. Fifty-three percent of patients developed distant metastases.
Recruitment	Early stage breast tumors or axillary lymph node metastases of triple-negative breast cancer patients were engrafted to obtain PDX models.
Ethics oversight	The protocol was approved by the Institut Curie Hospital committee (CRI: Comité de Revue Institutionnel) and by the Translational Research Board (Netherlands Cancer Institute).

Note that full information on the approval of the study protocol must also be provided in the manuscript.

Field-specific reporting

Please select the one below that is the best fit for your research. If you are not sure, read the appropriate sections before making your selection.

[x] Life sciences [ ] Behavioural & social sciences [ ] Ecological, evolutionary & environmental sciences

For a reference copy of the document with all sections, see nature.com/documents/nr-reporting-summary-flat.pdf

Life sciences study design

All studies must disclose on these points even when the disclosure is negative.

Sample size	Control and cisplatin/carboplatin treated groups included between 4 and 13 mice, with the exception of HBCx-157 for which 3 xenografts were treated. They were determinated based on previous experience with the PDX models and the intrinsic intra-tumor heterogeneity (Coussy et al. STM 2020; Romanelli et al. MCT 2012; Marangoni et al. CCR 2018).
Data exclusions	No data were excluded.
Replication	In vivo experiments were not replicated for logistic and ethical reasons. Biological replicates were included in each in vivo experiment (independent xenografts). Cell line experiments were replicated 3 times.
Randomization	Xenografts were randomly assigned in the different treatment groups at the beginning of each experiment. Allocation was random in cell line studies.
Blinding	Collection of data and analysis from in vitro and in vivo experiments were not blinded (treatment group are clearly indicated for each animal box). Analysis of shallow HRD status was blinded.

Reporting for specific materials, systems and methods

We require information from authors about some types of materials, experimental systems and methods used in many studies. Here, indicate whether each material, system or method listed is relevant to your study. If you are not sure if a list item applies to your research, read the appropriate section before selecting a response.

Materials & experimental systems

n/a	Involved in the study	
☐	☒	Antibodies
☐	☒	Eukaryotic cell lines
☒	☐	Palaeontology and archaeology
☐	☒	Animals and other organisms
☒	☐	Clinical data
☒	☐	Dual use research of concern

Methods

n/a	Involved in the study	
☒	☐	ChIP-seq
☒	☐	Flow cytometry
☒	☐	MRI-based neuroimaging

Antibodies

Antibodies used	Primary antibodies:<br>RAD51, anti mouse Genetex, GTX70230, Clone 14B4, Lot 44265 (IF 1:250)<br>Geminin, anti rabbit, ProteinTech Group, 10802-1-AP, Lot 00047193 (IF 1:500)<br><br>Secondary antibodies:<br>Goat anti-Mouse IgG (H+L) Cross Adsorbed Secondary Antibody, Alexa Fluor 647 (#A-21235, Invitrogen, 1;500)<br>Goat anti-Rabbit IgG (H+L) Cross Adsorbed Secondary Antibody, Alexa Fluor 488 (#A-11008, Invitrogen, 1;500)
Validation	RAD51 antibody was validated by running a Western blot on cell lines where RAD51 was knocked down using CRISPR/Cas9 sgRNAs, Geminin was validated by the manufacturer https://www.ptglab.com/products/GMNN-Antibody-10802-1-AP.htm

Eukaryotic cell lines

Policy information about cell lines and Sex and Gender in Research

Cell line source(s)	HEK293T cells were obtained from ATCC. RPE-1 cells were kindly provided by Daniel Durocher (PMID: 29973717).
Authentication	Cell lines were not authenticated.
Mycoplasma contamination	The cell lines were regularly tested negative for mycoplasma.
Commonly misidentified lines<br>(See ICLAC register)	No misidentified lines were used

Animals and other research organisms

Policy information about studies involving animals; ARRIVE guidelines recommended for reporting animal research, and Sex and Gender in Research

Laboratory animals	Swiss female nude mice were used for in vivo studies (8- to 12-week-old female).
Wild animals	the study did not involve wild animals
Reporting on sex	Female mice were used in this study.
Field-collected samples	Mice were maintained in specific pathogen-free animal housing.<br>Relative humidity of the mouse facility 30%-70% ; Temperature22°C +/-2°C ; Dark /light cycles: 12h/12h
Ethics oversight	Human breast tumor fragments were obtained with informed consent (Institute Curie) or approval of the Translational Research Board (Netherlands Cancer Institute). In vivo experimental procedures were approved by the Institutional Animal Care and French Committee (project authorization no. 02163.02) and Netherlands Cancer Institute animal experiments committee and were performed according to institutional regulations

Note that full information on the approval of the study protocol must also be provided in the manuscript.
