# Supplementary material for: Homologous recombination deficiency derived from whole-genome sequencing predicts platinum response in triple-negative breast cancers
Source: Nat Commun. 2023 Apr 7;14:1958. doi: 10.1038/s41467-023-37537-2 (PMC10082194; doi:10.1038/s41467-023-37537-2)
Supplement: Supplementary file 8 — Description of Additional Supplementary Files [file 41467_2023_37537_MOESM8_ESM.pdf]

Title: Supplementary data 1:

Description: molecular features of PDX models and patients' clinical characteristics.

Title: Supplementary Data 2:

Description: Whole exome sequencing analysis showing genomic mutations in the PDX panel.

Title: Supplementary Data 3:

Description: Fusion events affecting DDR genes in primary and PDX tumors. Fusion events detected in a panel of breast cancer PDX models using STAR-Fusion and filtered according to pipeline shown in Figure 1. Chromosomal position of left and right break according to Hg38. The column Fusion partners indicates the genes forming the left breakpoint of the fusion (APOPT1, GPX4) or the right breakpoint of the fusion (ZYVE21, DAZAP1).

Title: Supplementary data 4:

Description: cancer gene census (CGC) gene lists used for filtering of whole exome and RNA sequencing data.

Title: Supplementary Data 5:

Description: DNA damage repair (DDR) gene lists used for filtering of whole exome and RNA sequencing data.
